# Mitochondrial reactive oxygen species regulate acetyl-CoA flux between cytokine production and fatty acid synthesis in effector T cells

**DOI:** 10.1016/j.celrep.2025.115430

**Published:** 2025-03-13

**Authors:** Beibei Wu, Jin Seok Woo, Spyridon Hasiakos, Calvin Pan, Shawn Cokus, Cristiane Benincá, Linsey Stiles, Zuoming Sun, Matteo Pellegrini, Orian S. Shirihai, Aldon J. Lusis, Sonal Srikanth, Yousang Gwack

**Affiliations:** 1 Department of Physiology, David Geffen School of Medicine, University of California, Los Angeles, Los Angeles, CA 90095, USA; 2 Division of Oral Biology and Medicine, UCLA School of Dentistry, Los Angeles, CA 90095, USA; 3 Department of Medicine, Division of Cardiology, David Geffen School of Medicine, University of California, Los Angeles, Los Angeles, CA 90095, USA; 4 Department of Molecular, Cell and Developmental Biology, University of California, Los Angeles, Los Angeles, CA 90095, USA; 5 Department of Medicine, Endocrinology, David Geffen School of Medicine, University of California, Los Angeles, Los Angeles, CA 90095, USA; 6 Metabolism Theme, David Geffen School of Medicine, University of California, Los Angeles, Los Angeles, CA 90095, USA; 7 Department of Molecular and Medical Pharmacology, University of California, Los Angeles, Los Angeles, CA, USA; 8 Department of Immunology & Theranostics, Arthur Riggs Diabetes and Metabolism Research Institute, Beckman Research Institute of the City of Hope, Duarte, CA 91010, USA; 9 Molecular Cellular Integrative Physiology, University of California, Los Angeles, Los Angeles, CA 90095, USA; 10 Present address: Rheumatism Research Center, Catholic Research Institute of Medical Science, The Catholic University of Korea, Seoul 06591, South Korea; 11 These authors contributed equally; 12 Senior author; 13 Lead contact

## Abstract

Genetic and environmental factors shape an individual’s susceptibility to autoimmunity. To identify genetic variations regulating effector T cell functions, we used a forward genetics screen of inbred mouse strains and uncovered genomic loci linked to cytokine expression. Among the candidate genes, we characterized a mitochondrial inner membrane protein, TMEM11, as an important determinant of Th1 responses. Loss of TMEM11 selectively impairs Th1 cell functions, reducing autoimmune symptoms in mice. Mechanistically, *Tmem11*^−/−^ Th1 cells exhibit altered cristae architecture, impaired respiration, and increased mitochondrial reactive oxygen species (mtROS) production. Elevated mtROS hindered histone acetylation while promoting neutral lipid accumulation. Further experiments using genetic, biochemical, and pharmacological tools revealed that mtROS regulate acetyl-CoA flux between histone acetylation and fatty acid synthesis. Our findings highlight the role of mitochondrial cristae integrity in directing metabolic pathways that influence chromatin modifications and lipid biosynthesis in Th1 cells, providing new insights into immune cell metabolism.

## In brief

Using a forward genetics screen, Wu et al. uncovered the role of an inner mitochondrial membrane protein, TMEM11, in regulating Th1 cell effector functions. *Tmem11*^−/−^ Th1 cells show alterations in mitochondrial cristae integrity and increased mtROS levels that influence cellular metabolism and chromatin modifications, resulting in decreased effector functions.

## Graphical Abstract

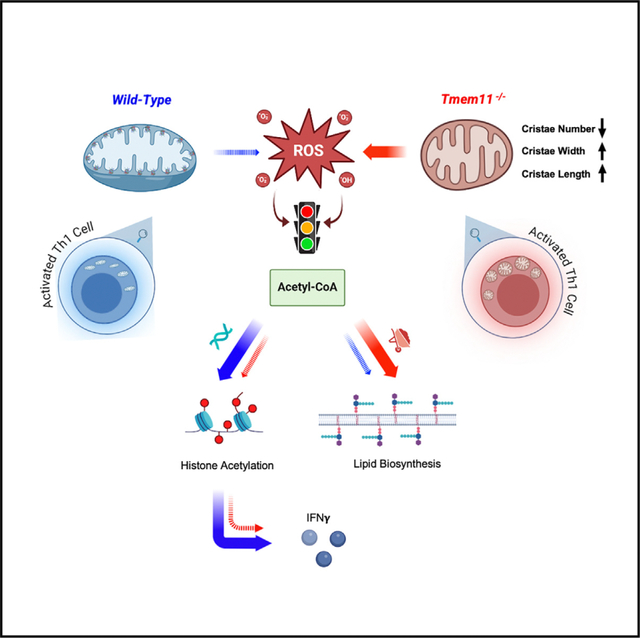

## INTRODUCTION

Effector helper T cells are major culprits of various autoimmune diseases, including type 1 diabetes, multiple sclerosis, rheumatoid arthritis, Crohn’s disease, and psoriasis. Therefore, a deeper understanding of mechanisms regulating the function of autoreactive T cells is needed to improve diagnostics and therapeutics of autoimmune diseases. Genome-wide association studies (GWASs) of human subjects demonstrated that genetic factors influence CD4^+^ T cell functions.^1–3^ However, extrinsic factors, including diet, climate, and microbiota, can complicate these studies. To specifically identify genetic factors influencing T cell functions, a hybrid mouse diversity panel (HMDP) provides many benefits.^4^ The HMDP consists of ~100 inbred strains, which have been densely genotyped and firmly established for mathematical models. Therefore, this method has been widely used to identify gene traits relevant to many complex diseases, including obesity, diabetes, atherosclerosis, fatty liver disease, and heart failure.^4,5^ Since these mice are housed under identical specific-pathogen-free conditions and fed the same diet, the HMDP provides an ideal forward genetic screening system by minimizing environmental variations.

T cells have dramatically different nutrient demands and metabolic programs during quiescence or differentiation.^6,7^ Quiescent naive and memory T cells rely on oxidative phosphorylation, whereas effector T cells are predominantly glycolytic, generating building blocks for cell growth and proliferation. Mitochondria are crucial in orchestrating these metabolic programs, including bioenergetics, metabolite supply, and signaling intermediates. Accordingly, the biogenesis and shape of mitochondria, including length and diameter, vary, with naive T cells showing elongated and more fused mitochondria, whereas effector T cells harbor rounded mitochondria.^6,7^ T cell-specific deletion of genes involved in mitochondrial biogenesis has been used to assess the role of mitochondrial architecture in T cells. However, whole-body deletion of these genes causes embryonic lethality in mice due to their essential and ubiquitous functions,^8–10^ raising concerns regarding the broad impact of their deletion on overall mitochondrial biology and cellular physiology. Hence, better genetic models that specifically impact mitochondrial function without grossly influencing cellular physiology are essential to unveil the specific role of mitochondria in effector T cell functions.

The electron transport chain (ETC) in the mitochondrial inner membrane also generates mitochondrial reactive oxygen species (mtROS) that can play both positive and negative roles in effector T cells depending on their level.^11–13^ T cell receptor (TCR) signaling leads to elevated intracellular Ca^2+^ concentration to drive the tricarboxylic acid (TCA) cycle and ETC, increasing the mitochondrial inner membrane potential and mtROS. Neutralization of mtROS antagonizes interleukin-2 (IL-2) production and expression of CD25 and CD69,^14^ whereas excessive mtROS cause poor production of cytokines, exhaustion, senescence, and death in T cells.^15,16^ In addition to generating energy and mtROS, the mitochondria also generate crucial metabolites that can influence epigenetic remodeling in the nucleus. Increased pyruvate influx via glycolysis to mitochondria enhances the generation of citrate, which is exported to the cytoplasm and converted to acetyl-coenzyme A (acetyl-CoA), which acts as a cofactor for histone acetylation.^17^ More recently, genetic evidence demonstrated a critical role for citrate export in T cell epigenetic reprogramming. Deletion of either the citrate transporter, Slc25a1, or ATP-citrate lyase (ACLY) in activated CD4^+^ T cells decreased H3K9 acetylation and *Ifng* transcription, emphasizing the role of mitochondria in T helper 1 (Th1) effector functions.^18^ Although these mitochondrial pathways are crucial for the effector T cell responses, the relationship between ROS signaling and acetyl-CoA availability for cytokine production remains unknown.

In this study, using a forward genetics screen, we identified TMEM11, a regulator for crista formation, as a crucial component of effector Th1 responses. Development and homeostasis of T cells were normal in *Tmem11*^−/−^ mice. However, while T cell differentiation was not affected, selective effector functions of Th1 cells were impaired due to the loss of TMEM11. This impairment decreased the severity of symptoms in an animal model of Th1 cell-mediated autoimmunity. Further, *Tmem11*^−/−^ Th1 cells showed abnormality in cristae architecture and mitochondrial respiration. Among these defects, excessive mtROS production by *Tmem11*^−/−^ Th1 cells channeled the flux of acetyl-CoA toward fatty acid (FA) synthesis, thereby reducing histone acetylation and cytokine production. Taken together, these studies delineate a selective role for mitochondrial inner membrane cristae structure in regulating Th1 effector functions.

## RESULTS

### Identification of TMEM11 as a genetic determinant of Th1 cell effector functions

The HMDP with 107 inbred strains was assembled from commercial vendors and new recombination strategies.^4,5^ Mice were housed in specific-pathogen-free conditions on a chow diet. Splenocytes from each strain were cultured in the presence of anti-CD3 antibodies alone or together with IL-6 + IL-23 and examined for cytokine production in CD4^+^ cells (Figure S1A). Effector T cells isolated from different strains showed a broad range of cytokine production. Among the strains with higher interferon-γ (IFN-γ)/IL-4 ratio was the SJL/J strain, which is widely used as an animal model for multiple sclerosis, while the well-known asthma-susceptible strain, BALB/cJ, belonged to the group of lower IFN-γ/IL-4 ratio. Two other strains, NZW/LacJ and AXB1/PgnJ, also behaved similar to BALB/cJ, showing a low IFN-γ/IL-4 ratio. However, these strains showed a clear difference in IL-17A expression. T cells from BALB/cJ and NZW/LacJ mice hardly expressed any IL-17A, while the ones from AXB1/PgnJ mice showed maximal IL-17A expression among all the strains tested. The genome-wide single-nucleotide polymorphism (SNP) analysis of these 107 strains based on cytokine production profile uncovered four genetic loci on chromosomes 1, 3, 11, and 18 to be associated in cis with cytokine expression (Figure S1B).

The identified loci encompass ~100 protein-coding genes, from which we selected candidates based on their abundance of expression in effector T cells (Figure S2A). The roles of some of these candidates, including transformation-related protein 53 binding protein 2 (Trp53bp2) and ubiquitin-specific peptidase 14 (Usp14), in effector T cells have been described.^19,20^ Among the novel candidates, TMEM11, a mitochondrial inner membrane protein, significantly influenced IFN-γ expression in murine T cells upon exogenous expression (Figure S2B). Interestingly, *TMEM11* depletion in human CD4^+^ cells cultured under Th1-polarizing conditions decreased IFN-γ expression (Figure S2C), indicating its conserved function between human and mouse T cells. TMEM11 is an inner mitochondrial membrane protein of ~21 kDa that was originally identified as a mitochondrial fission factor in *Drosophila* but was later shown to regulate mitochondrial crista length.^21,22^ Loss of the *Drosophila* homolog of TMEM11 did not influence fly development but increased the crista length in embryonic cells and neurons, resulting in impaired respiratory chain activity and increase in mitochondrial diameter and mtROS levels.^21^ Loss of TMEM11 in a mammalian cell line, U2OS, shows similar changes in crista length and mitochondrial diameter.^23^ Previously, the role of mitochondrial morphology in effector T cells has been examined using mice lacking expression of optic atrophy 1 (OPA1) or transcription factor A, mitochondrial (TFAM).^6,24^ However, *Opa1* or *Tfam* deletion can impact overall mitochondrial physiology, including their biogenesis. Therefore, we examined whether TMEM11 deletion would provide a specific genetic tool to determine the role of crista architecture in the function of effector T cells without gross influence on mitochondrial biology or cellular physiology.

### TMEM11 deficiency influences effector Th1 responses without affecting overall Th1 differentiation program

*Tmem11*^−/−^ mice showed no significant defect in body weight, survival, fertility, or lifespan. In addition, development and homeostasis of CD4^+^ and CD8^+^ T cells in the thymus and lymphoid organs were normal (Figures S3A and S3B). Expression of activation markers, including CD25 and CD69, in CD4^+^ and CD8^+^ T cells was normal in spleen and lymph nodes (LNs) of *Tmem11*^−/−^ mice (Figure S3C). Expression of CD62L and CD44, indicators of naive and memory T cells, in *Tmem11*^−/−^ CD4^+^ and CD8^+^ cells was similar to that in controls (Figure S3D).

**Figure 1.**
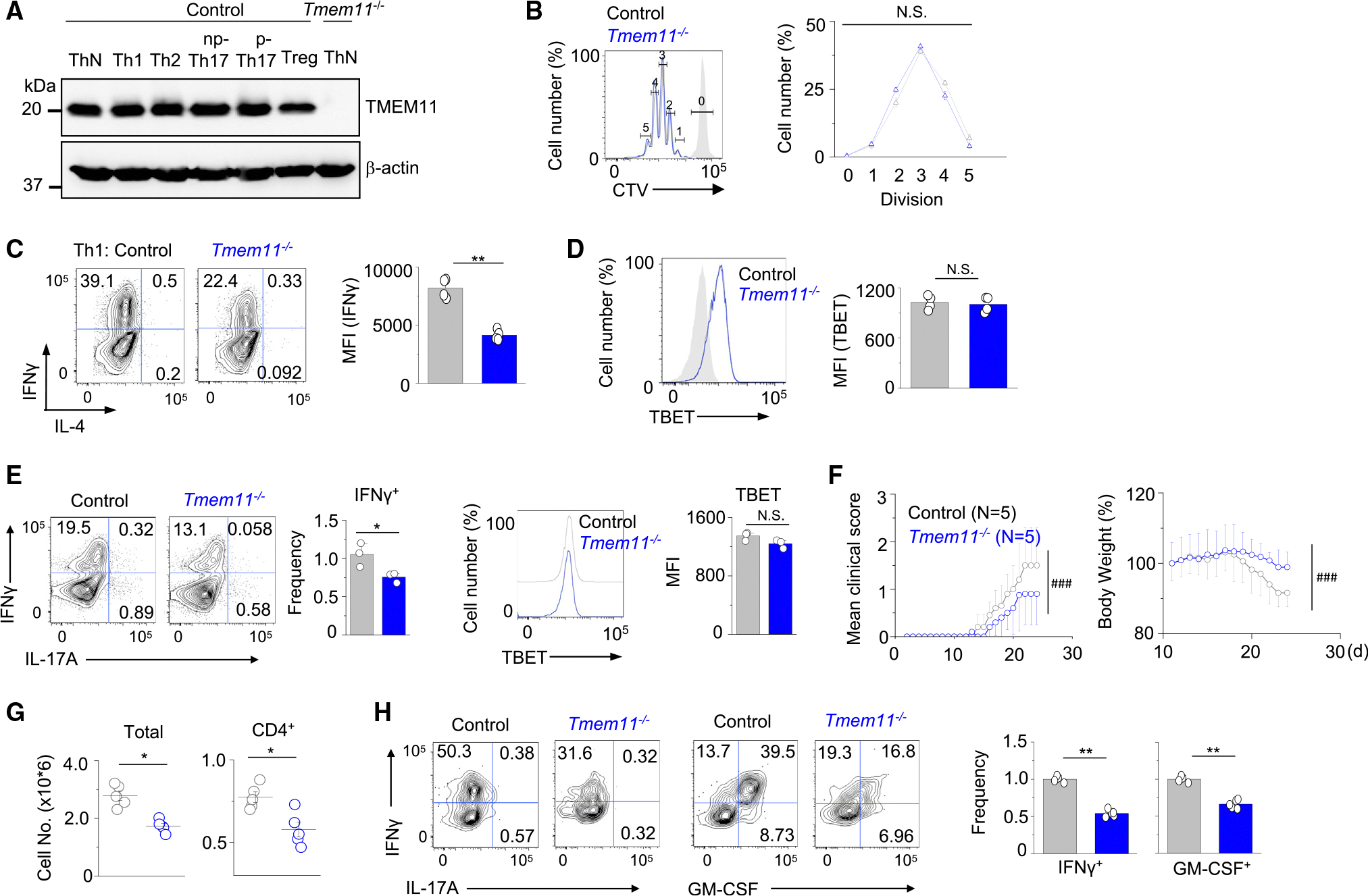
TMEM11 deficiency decreases the effector function of Th1 cells (A) Representative immunoblot showing expression of TMEM11 in effector T cells cultured under non-polarizing (ThN) and Th1-, Th2-, non-pathogenic (np-) Th17-, pathogenic (p-) Th17-, and inducible Treg (Treg)-polarizing conditions. β-actin, loading control. (B) Representative flow plots and line graphs showing cell trace violet (CTV) dilution rate in WT and *Tmem11*^−/−^ T cells cultured under Th1-polarizing conditions for 4 days. Unstimulated WT naive T cells served as control (light gray histogram). (C) Representative flow plots and bar graphs showing IFN-γ-expressing populations and mean fluorescence intensity (MFI) of IFN-γ in WT and *Tmem11*^−/−^ cells cultured under Th1-polarizing conditions. Cells were restimulated on day 4 with anti-CD3 and anti-CD28 antibodies for 5 h. (D) Representative flow plots (left) and bar graphs showing MFI of TBET expression (right) in WT and *Tmem11*^−/−^ cells cultured under Th1-polarizing conditions. WT T cells cultured under Th2-polarizing conditions were used as negative controls (gray, flow plot). (E) Representative flow plots and bar graphs showing IFN-γ and TBET expression in CD4^+^ cells from the draining lymph nodes of control or *Tmem11*^−/−^ mice injected with MOG peptide for EAE induction and cultured under Th1-expansion conditions for 72 h. (F) Time course of the mean clinical score (left) and body weight measurement (right) of EAE in *Rag2*^−/−^ recipients of control or *Tmem11*^−/−^ draining lymph node cells cultured under Th1-expansion conditions. The line graphs show mean ± SEM from the indicated number of animals from one representative experiment of a total of three experiments. (G) Scatterplots showing the numbers of total mononuclear (left) or CD4^+^ T cells (right) isolated from the CNS of recipients of control or *Tmem11*^−/−^ cells at the peak of EAE. (H) Representative flow plots showing the cytokine profile of CD4^+^ T cells from the CNS of recipients of control or *Tmem11*^−/−^ cells at the peak of the disease. Bar graphs show average (±SEM) of normalized frequency of IFN-γ^+^ and GM-CSF^+^ cells. Individual points in bar graphs in (C), (D), (E), (G), and (H) show data from independent animals. Representative immunoblots and graphs summarize results from at least three independent experiments except where stated otherwise. Data represent means ± SEM; significance was determined by unpaired two-tailed t test (B, C, D, G, and H) and Mann-Whitney *U* test (F). ^###^*p* < 0.0001, *p < 0.05, and **p < 0.005. N.S., not significant.

Immunoblotting showed ubiquitous expression of TMEM11 in all effector T cell subtypes and its absence in *Tmem11*^−/−^ T cells (Figure 1A). TMEM11 deficiency did not influence T cell proliferation rate or viability *in vitro* (Figure 1B). However, *Tmem11*^−/−^ Th1 cells showed a substantial reduction in IFN-γ expression (Figure 1C). The expression of the Th1 lineage-specifying transcription factor TBET was not altered in *Tmem11*^−/−^ T cells (Figure 1D). Interestingly, despite its ubiquitous expression, the differentiation and effector function of T cells cultured under non-pathogenic and pathogenic Th17-polarizing conditions were not influenced by TMEM11 deficiency, as judged by expression of IL-17A and RORγt (Figures S4A and S4B). Also, IL-4 and FOXP3 expression in *Tmem11*^−/−^ T cells cultured under Th2- and regulatory T cell (Treg)-polarizing conditions was not influenced (Figures S4C and S4D). In addition, the frequencies of FOXP3^+^ Treg populations were normal in the thymus, spleen, and LNs of *Tmem11*^−/−^ mice (Figure S4E). Since the loss of TMEM11 influenced IFN-γ expression in Th1 cells, we checked the effector function of other IFN-γ-expressing cells, including CD8^+^ cytotoxic lymphocytes (CTLs). Surprisingly, IFN-γ and IL-2 expression in CTLs was not influenced by TMEM11 deficiency (Figure S4F). Together, these results suggest that TMEM11 loss does not affect overall T cell development, homeostasis, or proliferation but selectively decreases IFN-γ expression in Th1 cells.

To check the physiological role of TMEM11 in T cells, we used an animal model of autoimmunity, experimental autoimmune encephalitis (EAE), in which both Th1 and Th17 cells are important for the onset and pathogenicity of the disease.^25^ Active EAE-induced *Tmem11*^−/−^ mice showed significant amelioration of clinical symptoms when compared to littermate controls (Figure S5A). To check T cell-intrinsic function of TMEM11, we performed adoptive transfer of MOG peptide-specific Th1 and Th17 cells into *Rag2*^−/−^ mice. Cells from the draining LNs of active EAE-induced control and *Tmem11*^−/−^ mice cultured under Th1-expansion conditions (with IL-12) showed a decrease in IFN-γ expression but similar levels of TBET, consistent with the results from *in vitro* differentiation (Figure 1E). *Rag2*^−/−^ recipients reconstituted with *Tmem11*^−/−^ Th1 cells displayed a slight delay in development of EAE symptoms (Figure 1F). Further, the disease severity, including mean clinical score and body weight loss, was significantly reduced in recipients of *Tmem11*^−/−^ Th1 cells. In support of these data, total mononuclear and CD4^+^ T cell numbers were reduced in the CNS of recipients of *Tmem11*^−/−^ T cells (Figure 1G). There was also a significant reduction in production of IFN-γ and the key pro-inflammatory cytokine, GM-CSF, by CNS-infiltrated *Tmem11*^−/−^ Th1 cells (Figure 1H). On the contrary, TMEM11 deficiency did not influence the functions of Th17 cells. Cells from the draining LNs of EAE-induced control and *Tmem11*^−/−^ mice cultured under Th17-expansion conditions (IL-1β + IL-23) showed similar expression of IL-17A and RORγt in agreement with the results from *in vitro* T cell culture (Figure S5B). In addition, the onset and severity of EAE were similar in *Rag2*^−/−^ recipients of control or *Tmem11*^−/−^ Th17 cells (Figure S5C). Accordingly, the numbers and cytokine production profiles of the CNS-infiltrated CD4^+^ cells from the CNS of the recipients were similar (Figures S5D and S5E). Interestingly, IFN-γ production by Th1-like cells converted from Th17 was not influenced by *Tmem11* loss, suggesting possible differences in the transcriptional program controlling IFN-γ expression in these cells compared to Th1 cells. Collectively, these data from *in vitro* T cell culture and EAE demonstrate an important role for TMEM11 in the physiological functions of Th1 but not Th17 cells.

### TMEM11 deficiency influences transcription of selective sets of pro-inflammatory cytokines in Th1 cells

**Figure 2.**
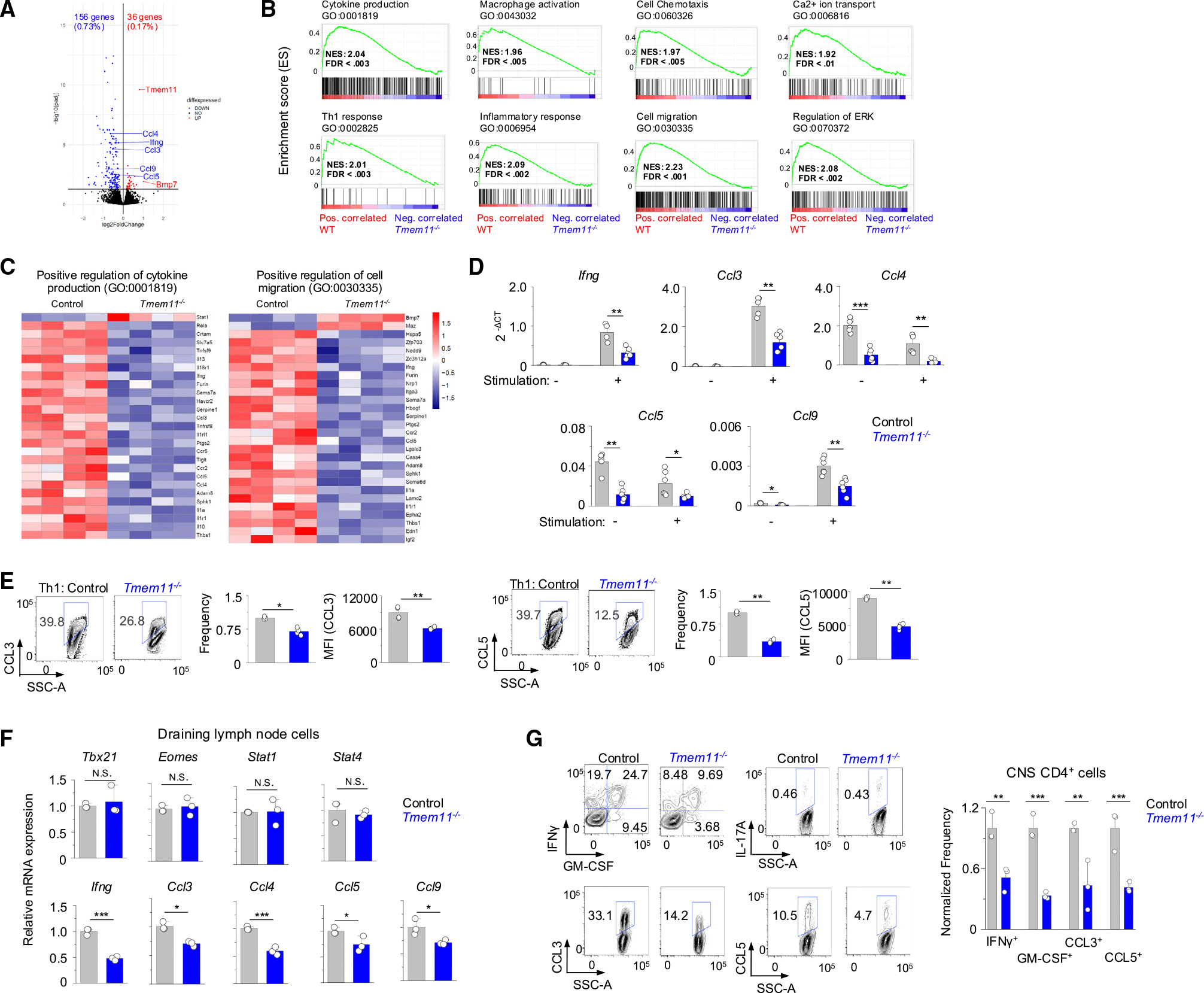
TMEM11 deficiency inhibits selective cytokine production in Th1 cells (A) Volcano plot of differentially expressed genes (DEGs) in control versus *Tmem11*^−/−^ cells cultured under Th1-polarizing conditions and restimulated with anti-CD3 and anti-CD28 antibodies for 5 h. Genes significantly (*p* adjusted < 0.01) upregulated and downregulated are depicted in red and blue, respectively. (B) Gene set enrichment analysis (GSEA) from control versus *Tmem11*^−/−^ Th1 cells. Phenotype correlation in WT (red) and *Tmem11*^−/−^ cells (blue) is shown. (C) Heatmaps showing *Z*-score-row-normalized expression values of genes within the indicated gene ontology sets in control versus *Tmem11*^−/−^ Th1 cells. (D) Transcript expression of indicated genes in control and *Tmem11*^−/−^ Th1 cells under resting conditions and 5 h after stimulation with anti-CD3 and anti-CD28 antibodies. Shown are pooled triplicates from two independent experiments. (E) Representative flow plots and bar graphs indicating frequency and MFIs showing expression of CCL3 and CCL5 in control and *Tmem11*^−/−^ Th1 cells. Each dot in the bar graphs represents data obtained from an independent experiment. (F) Transcript analysis of Th1-related transcription factors (top) and pro-inflammatory cytokines (bottom) in the cells from the draining lymph nodes of control *orTmem11*^−/−^ mice injected with MOG peptide for EAE induction after culturing under Th1-expansion conditions with IL-12 for 72 h. (G) Representative flow plots showing the cytokine profile of CD4^+^ T cells from the CNS of *Rag2*^−/−^ recipients of control or *Tmem11*^−/−^ cells at the peak of the disease (left). Bar graphs show averages (±SEM) of normalized frequency of cells expressing indicated cytokines (right). All experiments are representative of three biological replicates (unless otherwise indicated) with similar results. RNA-seq data were generated from experiments performed in triplicate. *p < 0.05, **p < 0.005, and ***p < 0.0005 (unpaired two-tailed t test).

To understand how TMEM11 influences Th1 function, we carried out transcriptome analysis of *in vitro*-differentiated control and *Tmem11*^−/−^ Th1 cells. This analysis showed a selective decrease in expression of gene sets involved in inflammation and chemotaxis by TMEM11 deficiency without influencing the overall Th1 transcriptional program, including lineage-specifying transcription factors (Figure 2A). Gene set enrichment analysis (GSEA) showed that TMEM11 deficiency selectively decreased expression of genes involved in Th1 responses, inflammation, and cell migration (Figure 2B). Interestingly, Ca^2+^ signaling and the extracellular signal-regulated kinase (ERK) pathway, which is regulated by Ca^2+^ signaling, appeared as one of the influenced signaling pathways due to TMEM11 deficiency.

Analysis of differentially expressed genes (DEGs) and Gene Ontology results revealed that transcription of the C-C motif chemokine ligand (CCL) family, including *Ccl3* (macrophage inflammatory protein [MIP]-1α), *Ccl4* (MIP-1β), *Ccl5* (RANTES), and *Ccl9* (MIP-1γ), was significantly reduced in *Tmem11*^−/−^ T cells (Figures 2A and 2C). To validate the RNA sequencing (RNA-seq results), we analyzed the expression of *Ccl3, Ccl4, Ccl5*, and *Ccl9* transcripts using qPCR. All were significantly reduced in *Tmem11*^−/−^ Th1 cells, similar to *Ifng* transcripts (Figure 2D). Interestingly, while transcripts of *Ccl3* and *Ccl9* were strongly induced by TCR restimulation, those of *Ccl4* and *Ccl5* were intrinsically highly expressed in Th1 cells regardless of TCR restimulation, suggesting their different regulatory mechanisms. These findings were further validated by intracellular staining, showing reduced CCL3 and CCL5 protein levels in *Tmem11*^−/−^ CD4^+^ cells cultured under Th1 conditions (Figure 2E).

Next, we examined the expression of these chemokines in the physiological setting of EAE. Cells from the draining LNs of active EAE-induced control and *Tmem11*^−/−^ mice cultured under Th1-expansion conditions showed decreased expression of *Ifng*, *Ccl3, Ccl4, Ccl5*, and *Ccl9* but similar expression of the lineage-specifying transcription factors (Figure 2F). CD4^+^ cells isolated from the CNS of *Rag2*^−/−^ recipients reconstituted with *Tmem11*^−/−^ Th1 cells also displayed reduced expression of CCL3, and CCL5, in addition to IFN-γ (Figure 2G). Collectively, these data show that loss of TMEM11 selectively influenced expression of pro-inflammatory cytokines and chemokines in Th1 cells.

### TMEM11 deficiency changes mitochondrial inner membrane architecture and respiration

**Figure 3.**
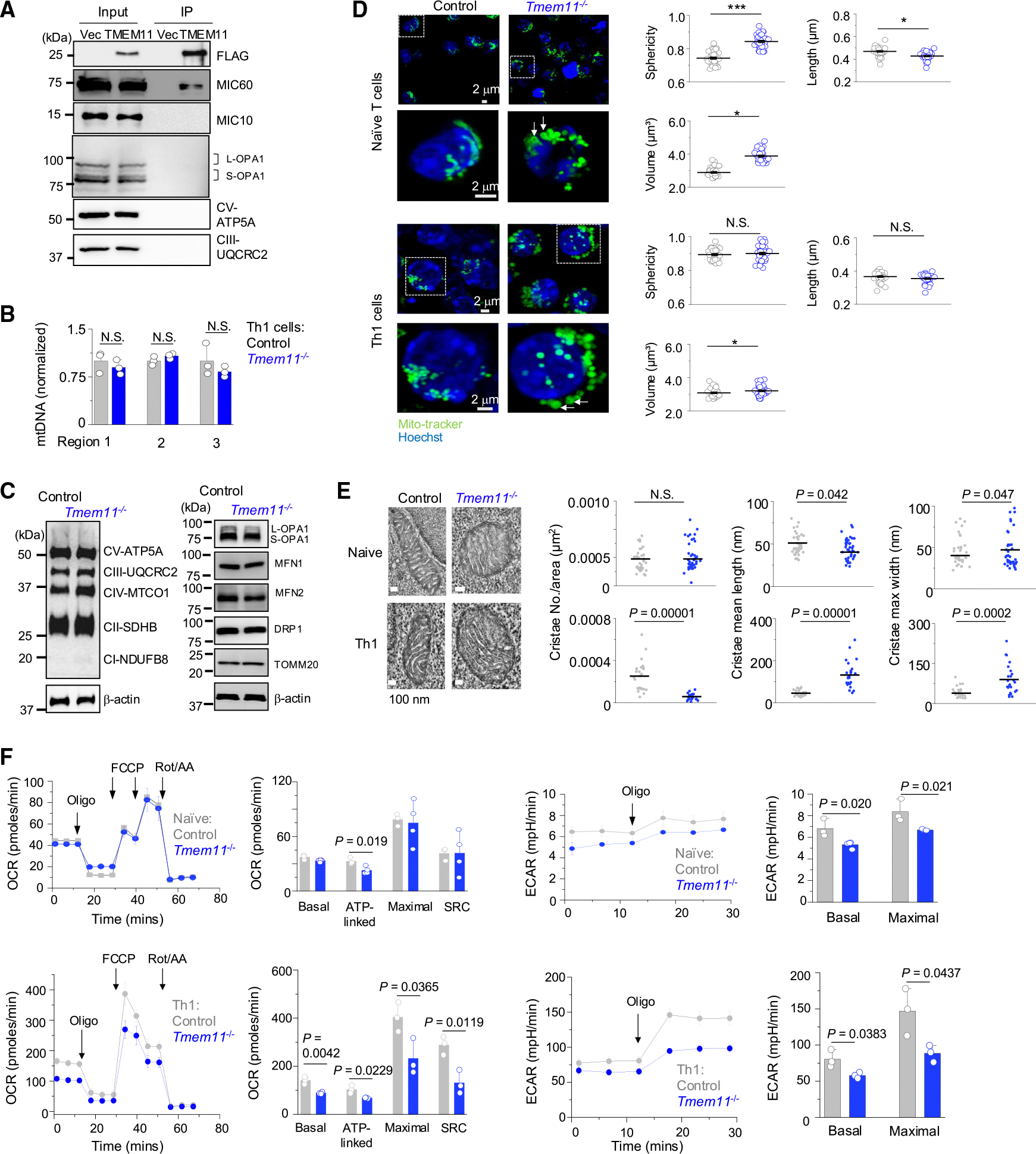
TMEM11 deficiency influences mitochondrial morphology, crista architecture, and respiration in effector T cells (A) FLAG-tagged TMEM11 expressed in *Tmem11*^−/−^ T cells cultured under Th1-polarizing conditions was immunoprecipitated with anti-FLAG antibody-conjugated resin and analyzed by immunoblotting for detection of indicated proteins. Vec, empty vector control; L-OPA1,long form; S-OPA1, short form. (B) Comparison of mitochondrial DNA content in WT and *Tmem11*^−/−^ cells cultured under Th1-polarizing conditions using quantitative PCR analysis of three different loci in the mitochondrial genome. (C) Representative immunoblots showing expression of ETC complex proteins and key mitochondrial membrane proteins in WT and *Tmem11*^−/−^ cells cultured under Th1-polarizing conditions. MFN, mitofusin; DRP1, dynamin-related protein 1; TOMM20, translocase of outer mitochondrial membrane 20. (D) Representative confocal images of WT and *Tmem11*^−/−^ naive T cells and effector T cells cultured under Th1-polarizing conditions stained with MitoTracker green and Hoechst (nucleus). Scale bar, 2 μm. White arrows indicate donut-shaped mitochondria. Scatterplots on the right show measurements of mitochondrial sphericity, length, and volume. Each symbol represents data from an independent cell. (E) Representative electron microscopy images of mitochondria from WT and *Tmem11*^−/−^ naive T cells (top) and Th1 cells (bottom) showing crista details. Scatterplots on the right show quantification of crista numbers, length, and maximal width from naive T cells (top) and Th1 cells (bottom). Each symbol represents data from an individual mitochondrion. (F) Oxygen consumption rate (OCR; left) and extracellular acidification rate (ECAR; right) measurements in control and *Tmem11*^−/−^ naive T cells (top) and effector T cells cultured under Th1-polarizing conditions (bottom). Th1 cells were stimulated with anti-CD3 and anti-CD28 antibodies for 2 h before analysis. Data are average ± SEM from cells cultured from three independent animals. All experiments are representative of three independent experiments (unless otherwise indicated) and graphs show means ± SEM from three independent experiments. *p < 0.05 and ***p < 0.0005 (unpaired two-tailed t test).

The mitochondrial inner membrane cristae organizing system (MICOS) complex, OPA1, and F_1_F_0_-ATP synthase (complex V) are essential for cristae formation.^26,27^ The MICOS complex, consisting of MIC60 and MIC10 sub-complexes, is involved in formation of the cristae junctions.^26,27^ MIC60 forms the mitochondrial intermembrane bridging complex with proteins at the outer membrane, while MIC10 establishes inner membrane curvature. The mitochondrial fusion protein OPA1 multimerizes with itself and maintains the width of cristae junctions. F_1_F_0_-ATP synthase localizes to the tip of the cristae to form membrane curvature. The other ETC complexes reside in the lateral surfaces of the cristae. Among these, TMEM11 was shown to interact with MIC60.^28^ To validate the role of TMEM11 as an interacting partner of the MICOS complex, we reconstituted *Tmem11*^−/−^ Th1 cells with FLAG-TMEM11 (Figure 3A). Using previously described buffer conditions containing 1% digitonin,^28^ we could detect MIC60 in anti-FLAG immunoprecipitates. This interaction was specific, since we could not detect other crista proteins, including MIC10, ETC complexes, and OPA1, under the same conditions.

Loss of the *Drosophila* TMEM11 ortholog decreased mitochondria number in neurons.^21^ Also, a recent report indicating the role of TMEM11 in mitophagy via interaction with BNIP3, an autophagy factor, suggests that TMEM11 deficiency may influence the number of mitochondria in T cells.^23^ Thus, we examined mitochondrial DNA (mtDNA) copy numbers in control and *Tmem11*^−/−^ Th1 cells. However, mtDNA copy numbers were similar between control and *Tmem11*^−/−^ Th1 cells (Figure 3B). Previous data had shown impaired expression of ETC components in *Tmem11*^−/−^ *Drosophila* cells.^22^ However, we observed similar expression of the ETC complexes as well as key mitochondrial membrane proteins in control and *Tmem11*^−/−^ Th1 cells (Figure 3C). Together, these results suggest that TMEM11 deficiency does not influence mitochondrial biogenesis or the amount of the ETC complex subunits in Th1 cells.

Loss of TMEM11 or its *Drosophila* homolog caused enlargement of mitochondria, suggesting its requirement for the maintenance of mitochondrial morphology.^21–23^ To examine mitochondrial morphology in *Tmem11*^−/−^ T cells, we performed confocal microscopy after MitoTracker green staining. Naive T cells from control mice showed a mixed population of elongated and rounded mitochondria, whereas those from *Tmem11*^−/−^ mice predominantly showed enlarged and rounded mitochondria with increased sphericity and volume but reduced length (Figure 3D, top). Effector T cells are known to harbor fragmented mitochondria.^29^ Accordingly, we observed rounded mitochondria in both control and *Tmem11*^−/−^ Th1 cells (Figure 3D, bottom). However, similar to naive T cells, *Tmem11*^−/−^ Th1 cells showed increased mitochondrial volume. Notably, both *Tmem11*^−/−^ naive and Th1 cells contained ring- or donut-shaped mitochondria (Figure 3D, white arrows), which are associated with mitochondrial swelling caused by the opening of the permeability transition pore or K^+^ channels, as well as mtROS production after treatment with complex I inhibitor, rotenone, or the complex III inhibitor antimycin.^30,31^

Electron microscopy (EM) analysis of neuronal cells from flies lacking a TMEM11 homolog showed increased crista length.^22^ Crista membranes also were highly elongated in a human cell line.^23^ To gain insight into the changes in crista architecture due to TMEM11 deficiency in T cells, we performed EM analysis of naive and Th1 cells. Mitochondria from *Tmem11*^−/−^ naive T cells showed a modest reduction in crista length and an increase in crista width when compared to those from wild-type (WT) T cells, while the number of cristae remained unchanged (Figure 3E, top). In contrast, mitochondria from *Tmem11*^−/−^ Th1 cells showed reduced numbers of cristae that were much longer and wider than those from control cells (Figure 3E, bottom). These results suggest that TMEM11 is important for determining mitochondrial crista length and width in Th1 cells, which can influence total mitochondrial volume, as previous findings strongly suggested a correlation between these two parameters.^22^

To determine the functional outcomes of changes in crista structure, we examined mitochondrial respiration in *Tmem11*^−/−^ T cells. *Tmem11*^−/−^ naive T cells show a modest reduction in ATP-linked oxygen consumption rate (OCR) and extracellular acidification rate (ECAR) compared with control cells (Figure 3F, top). Importantly, *Tmem11*^−/−^ Th1 cells showed pronounced impairment in basal, ATP-linked, maximal OCRs and ECAR (Figure 3F, bottom). Interestingly, mitochondrial respiration appears to depend more on crista architecture than global mitochondrial morphology, since impaired respiration was more pronounced in Th1 cells than in naive T cells. These data suggest that the decreased OCRs in *Tmem11*^−/−^ Th1 cells are a consequence of altered crista architecture rather than the overall increase in mitochondrial size. The mechanism underlying defective mitochondrial respiration in *Tmem11*^−/−^ Th1 cells is not clear at this point. However, considering similar ETC complex component expression in these cells (Figure 3C), it should be related to the optimal density of ETC complexes at the cristae.

### Excessive ROS generated by TMEM11 deficiency inhibit the effector function of Th1 cells

**Figure 4.**
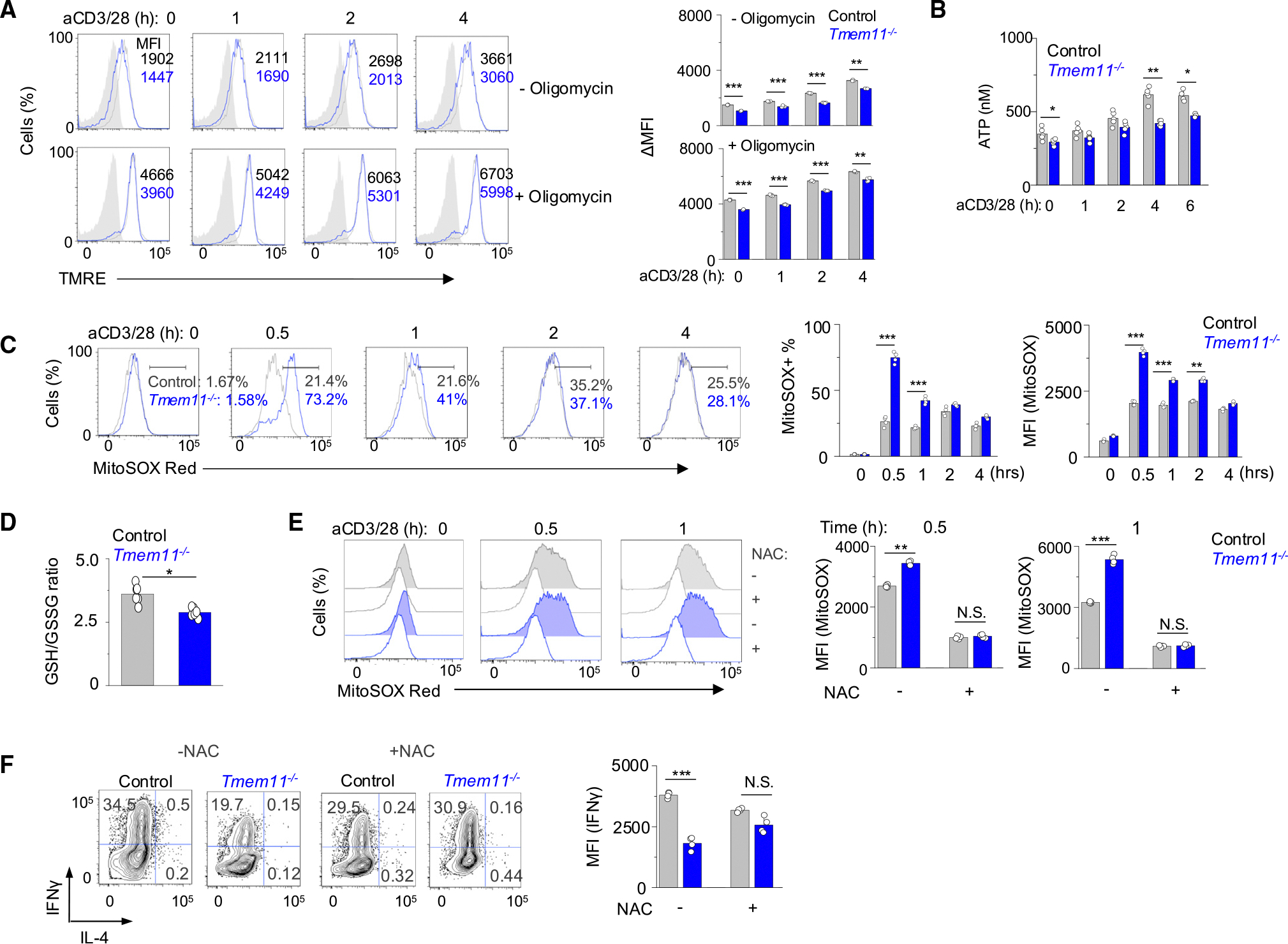
TMEM11 deficiency reduces Th1 effector cytokine production by increasing mitochondrial reactive oxygen species (A) Measurements of basal and oligomycin-induced maximal mitochondrial inner membrane potential in WT and *Tmem11*^−/−^ cells cultured under Th1-polarizing conditions and restimulated with anti-CD3 and anti-CD28 antibodies for indicated times. Bar graphs on the right show ΔMFI for tetramethylrhodamine-ethyl ester (TMRE) as calculated by subtracting the MFI values after carbonyl cyanide p-trifluoro-methoxyphenyl hydrazone (FCCP) treatment. Light gray flow plots show T cells with FCCP treatment. (B) Intracellular ATP levels in WT and *Tmem11*^−/−^ cells cultured under Th1-polarizing conditions. (C) Measurement of ROS levels in WT and *Tmem11*^−/−^ cells cultured under Th1-polarizing conditions after restimulation with anti-CD3 and anti-CD28 antibodies for the indicated times. (D) Cellular oxidative stress levels in WT and *Tmem11*^−/−^ cells cultured under Th1-polarizing conditions as determined by measuring the GSH/GSSH ratio after 2 h of restimulation with anti-CD3 and anti-CD28 antibodies. (E) Representative flow plots (left) and bar graphs (right) showing measurement of mtROS levels in WT and *Tmem11*^−/−^ cells cultured under Th1-polarizing conditions and treated overnight with 250 μM NAC and restimulated for indicated times in the presence of NAC. (F) Intracellular IFN-γ measurement in WT and *Tmem11*^−/−^ cells cultured under Th1-polarizing conditions, treated overnight with 250 μM NAC, and restimulated with anti-CD3 and anti-CD28 antibodies for 5 h in the presence of NAC. Bar graphs in (A)–(F) show means ± SEM from three independent experiments; significance was determined by unpaired two-tailed t test. *p < 0.05, **p < 0.005, and ***p < 0.0005.

In line with the decrease in OCR, the basal and maximal mitochondrial inner membrane potential was reduced in *Tmem11*^−/−^ Th1 cells (Figure 4A). Accordingly, we observed a modest decrease in ATP production by *Tmem11*^−/−^ Th1 cells (Figure 4B). Interestingly, we observed a striking increase in mtROS levels in *Tmem11*^−/−^ Th1 cells, especially at early time points after TCR stimulation (Figure 4C). Hence, we checked if excessive mtROS alter the antioxidant levels in *TMEM11*^−/−^ Th1 cells. The GSH/GSSG ratio in *Tmem11*^−/−^ Th1 cells was lower compared to WT cells, indicating increased oxidative stress in these cells due to high mtROS levels (Figure 4D).

OCR and ECAR were commonly reduced in other cell types, including non-pathogenic T17 cells, pathogenic Th17 cells, Th2 cells, and Treg cells (Figures S6A and S6B). However, a substantial increase in mtROS was observed only in *Tmem11*^−/−^ Th1 cells (Figure S6C). Furthermore, the GSH/GSSG ratio in non-pathogenic and pathogenic Th17 cells was very mildly influenced by the loss of TMEM11, unlike that in Th1 cells (Figure S6D). These results suggest that Th1 cells are intrinsically sensitive to high levels of mtROS possibly due to a weak antioxidant mechanism to reduce ROS levels. Importantly, treatment of *Tmem11*^−/−^ Th1 cells with a cell-permeative antioxidant, N-acetyl-L-cysteine (NAC), which decreased the cellular ROS levels, significantly rescued IFN-γ production (Figures 4E and 4F). These results suggest that excessive mtROS drive the decreased effector function of *Tmem11*^−/−^ Th1 cells.

### Excessive mtROS influence acetyl-CoA flux to inhibit the function of *Tmem11*^−/−^ Th1 cells

**Figure 5.**
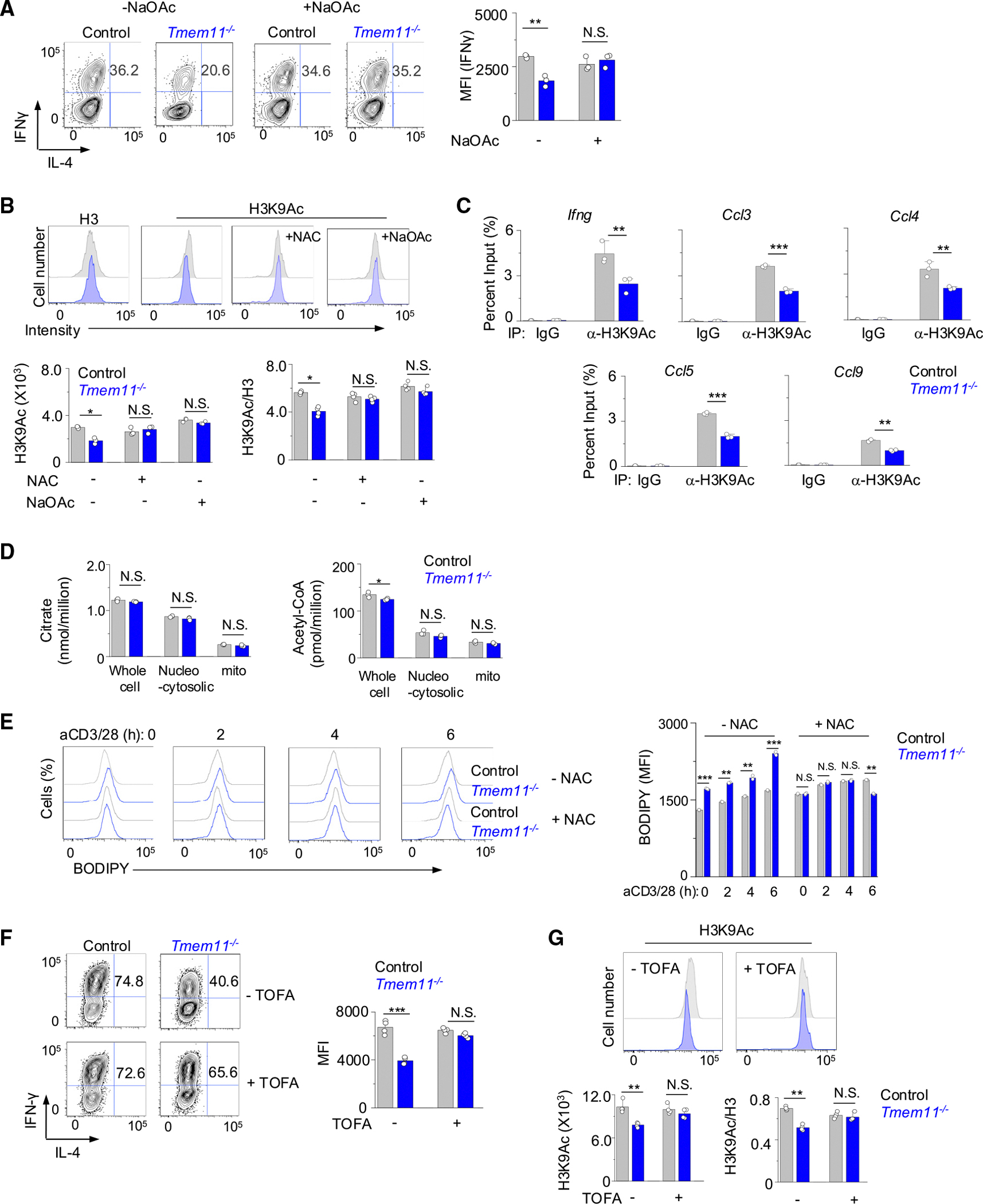
TMEM11 deficiency decreases H3K9Ac levels and increases fatty acid synthesis (A) Measurement of intracellular cytokine levels in WT and *Tmem11*^−/−^ cells cultured under Th1-polarizing conditions and treated with 20 mM sodium acetate (NaOAc) overnight and during restimulation with anti-CD3 and anti-CD28 antibodies for 5 h. (B) Measurement of H3K9 acetylation (H3K9Ac) levels in WT and *Tmem11*^−/−^ cells cultured under Th1-polarizing conditions. Cells were treated with 20 mM sodium acetate or 250 μM NAC overnight and during restimulation with anti-CD3 and anti-CD28 antibodies for 5 h. (C) Measurement of H3K9Ac levels of indicated gene loci in WT and *Tmem11*^−/−^ cells cultured under Th1-polarizing conditions using chromatin immunoprecipitation. Cells were restimulated with anti-CD3 and anti-CD28 antibodies for 5 h before nucleus collection. (D) Whole-cell, nucleocytoplasmic, and mitochondrial levels of citrate and acetyl-CoA in WT and *Tmem11*^−/−^ cells cultured under Th1-polarizing conditions. Th1 cells were restimulated with anti-CD3 and anti-CD28 antibodies for 5 h. (E) Representative flow plots (left) and bar graph showing measurement of neutral lipid levels in WT and *Tmem11*^−/−^ cells cultured under Th1-polarizing conditions and restimulated for the indicated times. (F) Measurement of intracellular cytokine expression in WT and *Tmem11*^−/−^ cells cultured under Th1-polarizing conditions and treated with 5 μM TOFA overnight and during restimulation with anti-CD3 and anti-CD28 antibodies for 5 h. (G) Measurement of cellular H3K9Ac levels in WT and *Tmem11*^−/−^ cells cultured under Th1-polarizing conditions and treated with 5 μM TOFA overnight and during restimulation with anti-CD3 and anti-CD28 antibodies for 5 h. Individual points in bar graphs in (A)–(G) show data from technical replicates, which are representative of at least two biological replicates. Data are shown as means ± SEM; significance was determined by unpaired two-tailed t test. **p < 0.005 and ***p < 0.005.

Increased pyruvate influx via glycolysis to mitochondria enhances the generation of citrate, which is exported to the cytoplasm and converted to acetyl-CoA, a cofactor for histone acetylation.^17^ Deleting either the citrate transporter Slc25a1 or ACLY in activated CD4^+^ T cells decreased H3K9 acetylation and *Ifng* transcription, emphasizing the role of mitochondria in this pathway.^18^ To check if cellular acetyl-CoA levels contributed to the phenotype of *Tmem11*^−/−^ T cells, we cultured control and *Tmem11*^−/−^ Th1 cells in the presence of sodium acetate (NaOAc), an exogenous source of acetyl-CoA, and checked IFN-γ expression. Interestingly, we observed a complete rescue of IFN-γ production in *Tmem11*^−/−^ Th1 cells cultured in the presence of NaOAc, while its effect was negligible in control cells (Figure 5A). Among acetylation of H3K4, H3K9, and H3K27, we found that H3K9Ac levels were selectively decreased in *Tmem11*^−/−^ Th1 cells, while methylation of H3K4 and H3K27 was not influenced (Figures S7A and S7B). H3K9Ac levels in *Tmem11*^−/−^ Th17 cells were not influenced (Figure S7C). Moreover, the addition of either NAC or NaOAc to *Tmem11*^−/−^ Th1 cells almost completely rescued acetylation of H3K9, indicating a negative impact of excessive mtROS on H3K9 acetylation (Figure 5B). We validated reduced H3K9Ac levels at the promoters of the *Ifng, Ccl3, Ccl4, Ccl5*, and *Ccl9* genes, which showed reduced expression in *Tmem11*^−/−^ Th1 cells, using chromatin immunoprecipitation (ChIP) analysis (Figure 5C).

We next checked whether altered crista architecture in *Tmem11*^−/−^ Th1 cells influences levels of TCA cycle metabolites. To our surprise, cellular concentrations of TCA metabolites, including citrate and acetyl-CoA, were similar between control and *Tmem11*^−/−^ Th1 cells (Figure 5D; Figures S8A and S8B). Next, we checked the possibility that the flux of acetyl-CoA toward histone acetylation was influenced by TMEM11 deficiency. Glucose-derived citrate can be exported into the cytosol to generate acetyl-CoA by ACLY for use in both protein acetylation and lipid synthesis.^32–34^ We found that WT Th1 cells showed progressively higher levels of neutral lipid upon stimulation, and its levels were higher in *Tmem11*^−/−^ Th1 cells under resting conditions, which increased further after stimulation (Figure 5E). Importantly, treatment of *Tmem11*^−/−^ Th1 cells with NAC reduced the neutral lipid levels to those similar to WT Th1 cells. To check if blocking the flux of acetyl-CoA toward lipid synthesis can rescue the histone acetylation and IFN-γ production defects in *Tmem11*^−/−^ Th1 cells, we cultured control and *Tmem11*^−/−^ cells in the presence of 5-tetradecyloxy-2-furoic acid (TOFA), an inhibitor of acetyl-CoA carboxylases (ACCs) that converts acetyl-CoA to malonyl-CoA, the substrate of FA synthase (FAS). Interestingly, blocking the FA synthesis pathway with TOFA treatment almost completely rescued the decrease in IFN-γ production and H3K9 acetylation in *Tmem11*^−/−^ Th1 cells, likely by increasing the availability of acetyl-CoA for histone acetylation (Figures 5F and 5G). C75, an inhibitor of FA synthase, also restored IFN-γ production and H3K9 acetylation in *Tmem11*^−/−^ Th1 cells with minimal effect on control cells (Figures S9A and S9B). Both treatments with TOFA and C75 decreased neutral lipid levels in *Tmem11*^−/−^ Th1 cells to those similar to control cells (Figure S9C). Together, these results suggest an important role for mtROS levels in determining the flux of acetyl-CoA to H3K9 acetylation versus FA synthesis.

### mtROS levels regulate acetyl-CoA flux to mediate H3K9 acetylation versus FA synthesis

**Figure 6.**
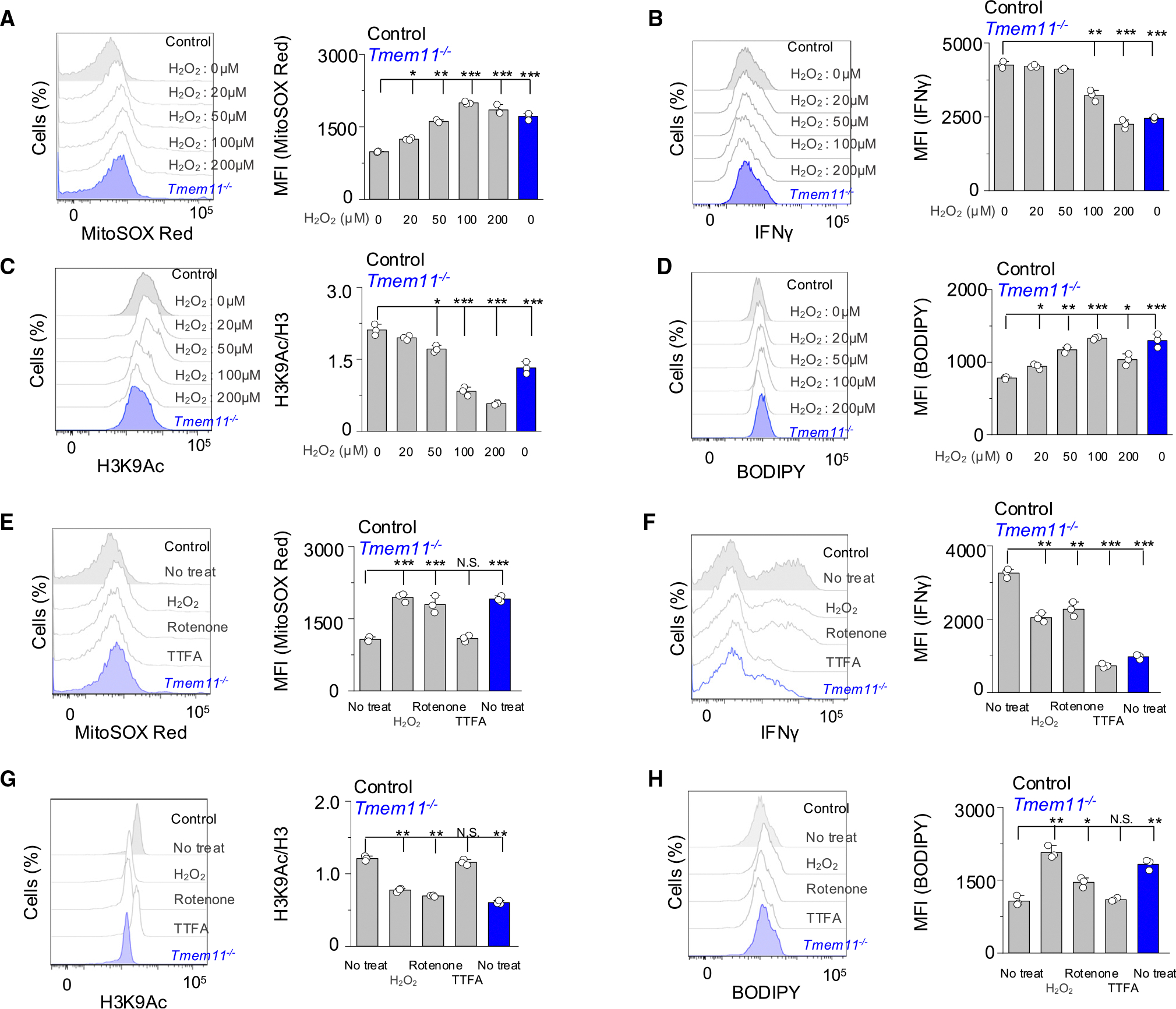
Excessive ROS are sufficient to decrease cellular H3K9 acetylation and increase fatty acid synthesis in Th1 cells (A–D) Measurement of ROS (A), intracellular cytokines (B), cellular H3K9Ac (C), and neutral lipids (D) in WT T cells cultured under Th1-polarizing conditions after treating cells with indicated concentrations of extracellular H_2_O_2_ during restimulation with anti-CD3 and anti-CD28 antibodies for 5 h. *Tmem11*^−/−^ Th1 cells without H_2_O_2_ treatment were used for comparison of phenotypes. (E–H) Measurement of ROS (E), intracellular cytokines (F), cellular H3K9Ac (G), and neutral lipids (H) in WT T cells cultured under Th1-polarizing conditions after inhibition of ETC complex I using 10 μM rotenone during restimulation for 5 h. Inhibition of ETC complex II using 100 μM TTFA was used as a negative control for ROS generation. *Tmem11*^−/−^ Th1 cells without rotenone treatment were used for comparison of phenotypes. Individual points in bar graphs in (A)–(H) show technical replicates from two independent experiments. Data are shown as means ± SEM. Significance was determined by unpaired two-tailed t test. **p < 0.005 and ***p < 0.005.

We next asked if increased ROS levels are sufficient to influence the flux of acetyl-CoA between histone acetylation and FA synthesis. Incubation of Th1 cells during restimulation with increasing concentrations of extracellular H_2_O_2_ showed an acute increase in cellular ROS in the presence of 100–200 μM H_2_O_2_ and accordingly decreased IFN-γ production, similar to that observed in *Tmem11*^−/−^ Th1 cells (Figures 6A and 6B). H_2_O_2_-treated Th1 cells also showed reduced H3K9 acetylation and increased neutral lipid levels, similar to those observed in *Tmem11*^−/−^ Th1 cells (Figures 6C and 6D).

We considered the possibility that an endogenous source of mtROS may better impact these pathways. Inhibition of ETC promotes mtROS production, with the complex I inhibitor rotenone enhancing mtROS production, whereas the complex II inhibitor 2-thenoyltrifluoroactone (TTFA) does not affect mtROS production.^35^ Inhibition of complex I during restimulation increased mtROS levels similar to H_2_O_2_-treated WT cells or untreated *Tmem11*^−/−^ Th1 cells, while inhibition of complex II did not influence mtROS levels (Figure 6E). Acute inhibition of complex I or complex II suppressed IFN-γ production in WT T cells cultured under Th1 conditions, consistent with a previous report demonstrating their crucial role in IFN-γ production (Figure 6F).^18^ Interestingly, inhibition of complex I, but not complex II, decreased acetylation of H3K9 and increased FA synthesis (Figures 6G and 6H). Collectively, these results suggest that increased mtROS due to altered crista architecture in *Tmem11*^−/−^ cells is sufficient to alter the flux of acetyl-CoA toward *de novo* FA synthesis for nutrient storage over histone acetylation for cytokine production.

## DISCUSSION

TMEM11 regulates the structure of mitochondria, specifically the length and width of cristae, by interacting with MIC60.^23,24,28^ MIC60 is a key part of the complex that shapes the crista junctions and connects with proteins on the mitochondrial outer membrane. Without a TMEM11 homolog, flies developed normally but had mitochondria with larger diameters and longer cristae.^21^ This change impaired the activity of the respiratory chain and increased mtROS levels. However, in U2OS human osteosarcoma cells, TMEM11 deficiency lengthened cristae without affecting respiration or mtROS, likely because these cells mainly use glycolysis for energy.^25,36^ Recent findings also suggest a role for TMEM11 in inhibiting the BNIP3/BNIP3L-driven mitophagy pathway,^25^ although the loss of TMEM11 in T cells did not alter the number of mitochondria, indicating a possibly minor role in mitophagy, at least in Th1 cells. Our research confirms that in Th1 cells, TMEM11 maintains its crucial role in shaping cristae and affects both respiration and mtROS levels, aligning with results from studies on PMI-deficient flies.^23,24^

Currently, little is known about the role of mitochondrial crista architecture in effector T cell function. One recent study examined the role of mitochondrial crista architecture among naive and effector T cells and found that Th17 cells uniquely harbor fused mitochondria with tightly packed cristae.^29^ Using OPA1-deficient mice, this study showed an important role for mitochondrial morphology and crista architecture in the effector functions of Th17 cells. However, *Opa1* deletion impaired proliferation in all effector T cell subtypes and increased cell death in Th1 and Treg cells,^29^ suggesting its broader impact on cell physiology. On the contrary, the current study showed that TMEM11 deficiency did not influence development, differentiation, or proliferation of effector T cells. Transcriptome analysis also showed a selective reduction in effector cytokines due to the loss of TMEM11 without influencing overall Th1 transcriptional programs. Interestingly, even though both OPA1 and TMEM11 were widely expressed across all effector T cell subtypes (Figure 1A),^29^ their loss impaired effector functions only in specific T cells, Th17 cells in the case of *Opa1* deletion and Th1 cells in the case of *Tmem11* deletion. Since mitochondrial respiration was commonly reduced in all the effector T cell types, it is reasonable to assume that Th1 cells are more susceptible to elevated mtROS, possibly due to intrinsically low levels of antioxidants.

Acetyl-CoA is used to generate energy, synthesize metabolic intermediates for growth and replication, and acetylate proteins.^36,37^ Among the more highly acetylated proteins are the histones,^38^ which connect mitochondrial functions to gene regulation. Mechanistically, excessive mtROS by TMEM11 deficiency disrupted the balance between histone acetylation and FA synthesis in the utilization of acetyl-CoA. We found multiple pieces of evidence to support our conclusion. First, we found that excessive mtROS production by TMEM11 deficiency decreased H3K9 acetylation while it increased FA synthesis. Accordingly, neutralization of mtROS rescued the decreased H3K9 acetylation and normalized the FA levels in *Tmem11*^−/−^ Th1 cells. Pharmacological inhibition of FA synthesis using TOFA and C75 also rescued both IFN-γ expression and H3K9 acetylation in *Tmem11*^−/−^ Th1 cells. Second, incubation of WT Th1 cells with exogenous H_2_O_2_ could recapitulate the phenotypes of *Tmem11* deletion by impairing H3K9 acetylation and IFN-γ expression and increasing FA synthesis. Third, increasing mtROS levels intrinsically using rotenone also recapitulated the phenotypes of *Tmem11* deletion. These data not only validate excessive mtROS levels as the primary cause of defects in *Tmem11*^−/−^ Th1 cells but also reveal its novel role in governing cellular decisions toward histone acetylation versus FA synthesis. While the impact of oxidative stress on epigenetic alteration is known,^39,40^ that on FA synthesis has yet to be clearly defined. We speculate that histone acetyltransferases may be more sensitive to the increased intracellular ROS levels than enzymes involved in FA synthesis, including ACLY, ACC, or FA synthase. A recent report suggested a role for lipids in compensating for oxidized mitochondrial damage.^41^ One interesting possibility is that the increased lipid levels in *Tmem11*^−/−^ Th1 cells may indicate an intrinsic defense mechanism to mitigate excessive mtROS levels.

The growing list of human disorders associated with mitochondrial structure underscores the urgent need for a detailed molecular comprehension of how the mitochondrial membrane organization influences various disease states. In this context, our research offers compelling evidence that crista architecture plays a critical role in defining effector T cell metabolism and functions. By genetically altering the structure of cristae, we observed significant changes in mtROS signaling. These alterations notably influence the use of acetyl-CoA, bifurcating its pathway between histone acetylation, which affects gene expression, and FA synthesis, essential for cellular structure and energy storage. Our findings provide a robust molecular framework for exploring the contributions of crista architecture to the intricate functions of effector T cells, laying the groundwork for future investigations into its role in health and disease.

### Limitations of the study

One limitation of this study is the lack of clarity regarding the Th1 specificity observed with TMEM11 loss, despite its ubiquitous expression across various effector T cell subtypes. Interestingly, although pathogenic Th17 cells produce IFN-γ, their functions remained intact both *in vitro* and *in vivo*. Future research focusing on differences in antioxidant responses between distinct effector T cell subtypes may provide insights into the Th1-specific role of TMEM11. Another limitation is that, while our study established a direct role for mtROS in regulating acetyl-CoA flux toward FA synthesis, we have yet to identify the precise mtROS-sensitive step involved in histone acetylation. One potential target is histone acetyltransferase; however, this hypothesis remains to be experimentally validated.

## RESOURCE AVAILABILITY

### Lead contact

Requests for further information and resources and reagents should be directed to and will be fulfilled by the lead contact, Yousang Gwack (ygwack@mednet.ucla.edu).

### Materials availability

All unique plasmids and/or cell lines generated in the study are available from the lead contact with a completed materials transfer agreement.

### Data and code availability

All data needed to evaluate the conclusions in the paper are present in the paper and/or the supplemental information. Genomic data have been deposited at GEO and are publicly available as of the date of publication. Accession numbers are listed in the key resources table. This study did not generate any custom code. Any additional information required to reanalyze the data reported in this paper is available from the lead contact upon request.

## STAR★METHODS

### EXPERIMENTAL MODEL AND STUDY PARTICIPANT DETAILS

#### Cell lines and cultures

HEK293T cells were grown in DMEM (Mediatech) supplemented with 10% (v/v) fetal bovine serum (Hyclone), 2 mM L-glutamine (Mediatech), 10 mM HEPES (Mediatech) and Penicillin/Streptomycin (Mediatech) at 37°C and 5% CO_2_.

#### Mice

*Tmem11*^−/−^ mice in C57BL/6NCrl genetic background (TCPR0855_ADGQ) were purchased from The Center for Phenogenomics (ON, Canada). 7–10-week-old age and sex matched control (*Tmem11*^+/+^) and *Tmem11*^−/−^ littermate animals were used for *in vitro* experiments. For passive EAE experiments, age matched donor and *Rag2*^−/−^ recipients (purchased from The Jackson Laboratory) were used. *Tmem11*^−/−^ mice were backcrossed to C57BL/6 (Jackson Laboratories) mice for at least 3 generations, and age and sex matched control (*Tmem11*^+/+^) and *Tmem11*^−/−^ littermate animals from F3 breeding colony were used for *in vivo* experiments. All mice were maintained in pathogen-free barrier facilities and used in accordance with protocols approved by the Institutional Animal Care and Use Committee at the UCLA.

#### Human peripheral blood mononuclear cell culture

Peripheral blood mononuclear cells (PBMCs) were obtained under federal and state regulations from the CFAR Virology core Laboratory at UCLA that were prepared from buffy coats from healthy, unidentified adult donors using Ficoll-PAQUE gradients. Naïve CD4^+^ T cells were enriched by magnetic sorting from single-cell suspensions using MagniSort naïve CD4^+^ T cell enrichment kit according to manufacturer’s instructions (ThermoFisher Scientific). For effector T cell differentiation, cells were stimulated for 48h on a plate coated with 10 μg/ml of anti-CD3 antibody (OKT3, Bio X Cell). T cells were cultured in T cell media (DMEM containing 20% fetal bovine serum and 1% Pen-Strep) supplemented with 5 μg/ml of anti-CD28 antibody (Bio X cell), 10 ng/ml IL-12, and 20 U/ml IL-2 (Peprotech) for Th1 differentiation.

### METHOD DETAILS

#### Plasmids

Mouse T cell cDNA was used for PCR amplification of TMEM11 using primers described in Table S1 and cloned into pMSCV-CITE-eGFP-PGK-Puro vector with a C-terminal FLAG tag and expressing GFP from an IRES site.

#### Mouse panel screening

Mice (6–10 weeks of age) were housed in specific pathogen-free conditions on a chow diet. Splenocytes were isolated from 3–4 mice of 120 inbred mouse strains and cultured under non-polarizing (only anti-CD3 antibodies) or Th17-polarizing conditions (anti-CD3 antibodies together with IL-6 [30 ng/ml] and IL-23 [10 ng/ml]) to check cytokine levels (the ratio of IFNγ to IL-4 and IL-17A) in CD4^+^ cells by intracellular staining. Splenocytes were cultured for 3 days and re-stimulated with PMA (80 nM) plus ionomycin (1 μM) for 4 h prior to staining. Genotypes of all 107 mouse strains were obtained from the Jackson Laboratory using the Mouse Diversity Array. Single nucleotide polymorphisms (SNPs) that had poor quality or minor allele frequency of < 5% and a missing genotype rate of >10% were removed. After filtering, 200,000 SNPs were left. Genome-wide association was performed using Factored Spectrally Transformed Linear Mixed Models, and a cut-off p-value for genome-wide significance was set at 3.46 × 10^−6^. Locus plot was generated for genome-wide significant association using the standalone version of the LocusZoom package.^42^

#### RNA isolation, cDNA synthesis and real-time quantitative PCR

Total RNA from cells harvested in TRIzol Reagent (Thermofisher Scientific) was isolated using the Direct-zol RNA isolation kit (Zymo Research). RNA quantity and quality were confirmed with a NanoDrop ND-1000 spectrophotometer. cDNA was synthesized using 1–2 μg of total RNA using qScript cDNA SuperMix (Quantabio). Real-time PCR was performed using PerfeCTa qPCR SuperMix (Quantabio) on an iCycler IQ5 system (Bio-Rad) using gene-specific primers described in Table S1. Threshold cycles (C_T_) for all the candidate genes were normalized to those of 36B4 to obtain ΔC_T_. The specificity of primers was examined by melt-curve analysis and agarose gel electrophoresis of PCR products.

#### Confocal microscopy and electron microscopy

For confocal analysis, naïve CD4^+^ T Cells or Th1 cells were stained with 50 nM Mito-tracker green in Opti-MEM medium at 37°C for 30 mins, following by Hoechst 33342 staining for 10 mins. Cells were kept in a humidified incubation chamber at 37°C with 5% CO_2_ during image collection. Samples were imaged on a Zeiss LSM 880 confocal microscope used in Airyscan mode and processed using Zen Black software (Zeiss). For mitochondria morphology analysis, 3D confocal images were analyzed using the machine learning Aivia v.10 software (Leica Microsystems). Labelled mitochondria (MitoTracker green) were used for training of machine learning by pixel classification. Mitochondria were segmented and measured in the cellular 3D volume. For electron microscope imaging, naïve CD4^+^ T Cells or Th1 cells were washed and resuspended in serum-free RPMI and fixed in 2.5% glutaraldehyde in 100 mM sodium cacodylate buffer for 20 mins, and washed thrice in cacodylate buffer. Subsequently, samples were embedded in 4% agarose gel and post-fixed in 1% osmium tetroxide. After wash, samples were dehydrated through a graded series of ethanol concentrations and propylene oxide. After infiltration with Eponate 12 resin, the samples were embedded in fresh Eponate 12 resin and polymerized at 60°C for 48 h. Ultrathin sections of 70 nm thickness were prepared and placed on formvar carbon coated copper grids and stained with uranyl acetate. The grids were examined using a JEOL 100CX transmission electron microscope at 60 kV and images were captured by an AMT digital camera (Advanced Microscopy Techniques Corporation, model XR611) (Electron Microscopy Core Facility, UCLA Brain Research Institute). For TEM image analysis, correction of pixel intensities and the creation of RGB images was needed for proper segmentation by pixel classification using Aivia v.10 software. For that, ImageJ was used to subtract background (rolling 500 light sliding) and enhance local contrast (CLAHE: blocksize; 50, histogram: 256 maximum: 3) with two images average. RGB image was created from averaged image and pixel classification was created for dark pixels (cristae or mitochondria), medium dark pixels (cytoplasm) and light pixels (background). Segmentation was used for measurements of mitochondria in whole cells or cristae analysis in individually cropped mitochondria.

#### Immunoblotting and immunoprecipitation

For endogenous TMEM11 expression, naïve CD4^+^ T cells isolated from control and *Tmem11*^−/−^ mice were differentiated into various effector T cells as described below. 4 days after differentiation, cells were stimulated with anti-CD3 plus anti-CD28 antibody and stained for signature cytokines to validate appropriate differentiation. 10 million differentiated effector T cells were then lysed in RIPA buffer (10 mM Tris-Cl pH 8.0, 1% Triton X-100, 0.1% SDS, 140 mM NaCl, 1 mM EDTA, 0.1% sodium deoxycholate and protease inhibitor cocktail [Roche]) and centrifuged to remove debris. Samples were separated on 12% SDS-PAGE. Proteins were transferred to nitrocellulose membranes and subsequently analyzed by immunoblotting with anti-Tmem11 antibody. Polyclonal rabbit antibody for detection of TMEM11 was used at 1: 5,000 dilution. Anti-β-actin antibody was purchased from Santa Cruz Biotechnology (clone I-19), Chemiluminescence images were acquired using an Image reader LAS-3000 LCD camera (FujiFilm). For immunoprecipitation, cDNAs encoding FLAG-tagged TMEM11 were transfected into *Tmem11*^−/−^ Th1 cells. Transfected cells (1 × 10^7^) were lysed in cell lysis buffer (50 mM Tris–HCl [pH 7.5], 150 mM NaCl, 1% Digitonin, and protease inhibitor mixture) for 30 min on ice to obtain whole cell extracts^28^ before preclearing with protein G-Sepharose. Lysates were immunoprecipitated with anti-FLAG antibody-conjugated resin for 6 h. Immunoprecipitates were washed six times in cell lysis buffer and analyzed by immunoblotting. For immunoblot analyses cells were lysed in RIPA buffer (10 mM Tris-Cl pH 8.0, 1% Triton X-100, 0.1% SDS, 140 mM NaCl, 1 mM EDTA, 0.1% sodium deoxycholate and protease inhibitor cocktail [Roche]) and centrifuged to remove debris. Samples were separated on 8–12% SDS-PAGE. Proteins were transferred to nitrocellulose membranes and subsequently analyzed by immunoblotting with relevant antibodies. Chemiluminescence images were acquired using an Image reader LAS-3000 LCD camera (FujiFilm).

#### T cell purification, differentiation, stimulation, and staining

T cell purification, activation and differentiation were carried out as previously described.^43,44^ Briefly, naïve CD4^+^ T cells were enriched by magnetic sorting from single-cell suspensions generated by mechanical disruption of spleens and lymph nodes of adult mice using MagniSort naïve CD4^+^ T cell enrichment kit (catalog # 8804–6824-74, ThermoFisher Scientific). For effector T cell differentiation, cells were stimulated with 1 μg/ml of anti-CD3 antibody (1452C11, Bio X Cell) and 1 μg/ml of anti-CD28 antibody (Clone 37.51, Bio X Cell) for 48 h on a plate coated with 0.3 mg/ml of goat anti-hamster antibody (MP Biomedicals). CD4^+^CD25T^−^ cells were cultured without any polarizing cytokines or antibodies for non-polarizing (ThN) conditions, with 10 mg/ml anti-IL-4 antibody (Peprotech) and 2 ng/ml IL-12 for Th1 differentiation; 20 μg/ml anti-IFN-γ antibody (Bio X Cell), 2.5 μg/ml anti-IL-12 antibody and 10 ng/ml IL-4 for Th2 differentiation; 10 μg/ml anti-IL-4 antibody, 10 μg/ml anti-IFN-γ antibody, 30 ng/ml IL-6 (Peprotech) and 1 ng/ml TGF-β (Peprotech) for non-pathogenic Th17 differentiation; 10 μg/ml anti-IL-4 antibody, 10 μg/ml anti-IFN-γ antibody, 30 ng/ml IL-6 (Peprotech), 10 ng/ml IL-23 (R&D Systems) and 10 ng/ml IL-1β (R&D Systems) for pathogenic Th17 differentiation and cultured in T cell medium as described above. Cells were taken off the plate on D2 and cultured for 2 more days with additional medium. On day 4, differentiated T cells were re-stimulated with 1 μg/ml of anti-CD3 antibody and 1 μg/ml of anti-CD28 antibody for 5 h for cytokine analysis. Brefeldin A (1 μg/ml) was added for the last 2 h. Cells were harvested, stained with Fixable Viability Dye eFluor 780 (eBioscience) for 10 min in PBS on ice, washed in PBS, permeabilized with 0.5% saponin, and stained intracellularly for indicated cytokines. For staining of transcription factors, differentiated effector T cells were fixed/permeabilized and stained using Transcription Factor Staining Buffer Set (BD Pharmingen). For surface staning, 1 × 10^6^ cells from single cell suspensions of thymi, spleens and lymph nodes were stained with indicated antibodies in PBS + 1% fetal bovine serum, at 4°C for 20 mins, washed and used for data acquisition immediately. For CTV Labeling experiment, naïve T cells were labeled using CellTrace^™^ Violet Cell Proliferation Kit according to the manufacturer’s protocols. CTV-labeled control and *Tmem11*^−/−^ naïve T cells were cultured under Th1-polarizing conditions and were harvested and analyzed by flow cytometry on day 4. Data were acquired using BD LSRFortessa cell analyzer and analyzed using FlowJo software (Tree Star).

#### Metabolism assay

Naïve CD4^+^ T cells (8.0 × 10^5^ per well) and *in vitro* cultured Th1 cells (4.0 × 10^5^ per well) were plated on poly-D-Lysine-coated Seahorse culture plates in Seahorse XF DMEM medium (Agilent) supplemented with 2 mM L-glutamine (Gibco), 1 mM sodium pyruvate (Sigma) and 10 mM D-glucose (Sigma) and analyzed using a Seahorse XFe96 Bioanalyzer (Agilent). To check mitochondrial respiration, basal oxygen consumption was measured followed by the addition of 2 mM oligomycin (Cayman Chemicals), an ATP synthase inhibitor, 0.75 μM of the protonophore carbonyl cyanide-4-(trifluoromethoxy)-phenylhydrazone (FCCP, Cayman Chemical) and 1.0 μM rotenone (AdipoGen) together with 2.0 μM antimycin A (Sigma). The basal oxygen consumption was calculated by subtracting the OCR after rotenone and antimycin A treatment from the OCR before oligomycin treatment. The maximal OCR was calculated by subtracting the OCR after rotenone and antimycin A treatment from the maximal OCR measured after addition of FCCP.

#### Detection of cellular acetyl-CoA, citrate concentrations, ATP levels, GSH/GSSG ratio and metabolomics assay

Acetyl-CoA and citrate concentrations in different subcellular fractions were measured by the acetyl-coenzyme A assay kit (Sigma) and citrate assay kit (Sigma). 5×10^6^ Th1, npTh17 or pTh17 cells were harvested in 100 μl fractionation buffer (20 mM HEPES, 10 mM KCl, 2 mM MgCl2, 1 mM EDTA, 1 mM EGTA and 1 M dithiothreitol).^45^ Samples were incubated on ice for 30 min, followed by centrifugation at 10,000 g at 4°C for 2 min to separate nucleo-cytosolic fractions in the supernatant from the mitochondria in the pellet. Mitochondrial lysates were obtained by washing the pellet with ice-cold PBS and permeabilization with 80% methanol for 15 min on ice followed by centrifugation at 14,000 g for 1 min at 4°C. Intracellular ATP levels of Th1 cell on day 4 was determined by ATPlite 1step Luminescence Assay kit (PerkinElmer) following the manufacturer’s instructions. Th1 cells from control and *Tmem11*^−/−^ mice cultured for 4 days *in vitro* were examined for the ratio of total glutathione to oxidized glutathione using GSH/GSSG-Glo^™^ Assay kit (Promega). For metabolite tracing experiments, D4 Th1 cells were re-stimulated with anti-CD3 and anti-CD28 antibodies in medium containing ^13^C-Glucose for 5 h. Cells were washed once with 150 M ammonium acetate solution (pH 7.4) and then extracted in 1 ml 80% methanol (−80C), for 30 mins followed by centrifugation at 16,000 g for 10 min at 4C. The supernatant was dried and stored at −80°C until LC-MS analysis. Metabolites were detected with a Thermo Scientific Q Exactive mass spectrometer run with polarity switching in full scan mode with a m/z range of 70–975 and 70.000 resolution. Relative amounts of metabolites were calculated by summing up the intensities of all isotopologues of a given metabolite. C13 natural abundance corrections were made using AccuCor. Data analysis was performed using in-house R scripts.

#### Glucose uptake, BODIPY staining and ROS measurements

Glucose uptake by CD4^+^ T cells *in vitro* was monitored at indicated times using the fluorescent glucose analog 2-NBDG (Cayman Chemicals). After starving the cells for 15 min in glucose-free medium, T cells were incubated with 50 μM 2-NBDG for 30 min at 37°C, washed and processed for flow cytometric analysis. Neutral lipid content of Th1 cells under resting conditions or at indicated times after stimulation were measured using 2 μM BODIPY 493/503 reagent (Invitrogen) using manufacturer’s instructions. ROS production was measured using 2.5 mM MitoSOX Red (Invitrogen).

#### Measurement of mitochondrial membrane potential

For Measurement of mitochondrial membrane potential, effector CD4^+^ T cells were stimulated with 1 μg/ml of anti-CD3 antibody and 1 μg/ml of anti-CD28 antibody (Pharmingen) for indicated times on a plate coated with 0.3 mg/ml of goat anti-hamster and then cells were loaded with 15 nM Tetramethylrhodamin-Ethylester (TMRE) (Invitrogen). To check the background or maximum levels for TMRE, T cells were pre-treated with 2 μM Trifluoromethoxy carbonylcyanide phenylhydrazone (FCCP, Cayman Chemicals) or 2μM oligomycin for 15 min at 37 °C. Samples were acquired with a BD LSRFortessa (BD Biosciences) and analyzed with FlowJo (TreeStar).

#### Histone staining

To assess histone modifications, stimulated T cells were fixed with 4% PFA, permeabilized with ice-cold methanol and stained with anti-mouse acetyl-histone H3K4ac FITC (Abcam, EPR16596), anti-mouse acetyl-histone H3K9ac FITC (Cell Signaling, clone C5B11), anti-mouse histone H3 PE (Cell Signaling, clone D1H2), anti-mouse H3K27ac (Cell Signaling, clone D5E4) or rabbit monoclonal anti-mouse H3K27me3 (Cell Signaling, clone C36B11), anti- mouse H3K4me3 (Cell Signaling, clone D1A9) for 1h in permeabilization buffer (eBioscience). All sample acquisition was performed with a BD Celesta flow cytometer (BD Biosciences) or an Aurora Flow Cytometer (Cytek) and further analyzed with the FlowJo software (Tree Star).

#### EAE induction and analyses

Mice were immunized subcutaneously on day 0 with 100 μg of MOG_35–55_ peptide (N-MEVGWYRSPFSRVVHLYRNGK-C, Genscript) emulsified in complete Freund’s adjuvant (CFA, Difco) supplemented with 5 mg/mL of Mycobacterium tuberculosis H37Ra (Difco) as previously described.^44^ The mice were also injected i.p. with pertussis toxin (200 ng/mouse, List Biological Laboratories) on days 0 and 2. For passive EAE, donor control *Tmem11*^+/+^ and *Tmem11*^−/−^ mice were first immunized with MOG_35–55_ subcutaneously as described above. At the first sign of EAE symptoms (score 0.5), draining lymph node cells were collected and cultured under Th1 (IL12, 10 ng/ml) or Th17-expansion conditions (IL-23, 10 ng/mL and IL-1β, 10 ng/ml) together with 20 ug/ml of MOG_35–55_ peptide, for 3 days. At day 3, 5 × 10^6^ CD4^+^ T cells were injected intravenously into *Rag2*^−/−^ mice. EAE severity was scored according to the following clinical scoring system: 0, no clinical signs; 1, paralyzed tail; 2, partial hind leg paralysis; 3, complete hind leg paralysis or partial hind and front leg paralysis; 4, complete hind and partial front leg paralysis; 5, complete hind and front leg paralysis (moribund). When a mouse was euthanized because of severe paralysis, a score of 5 was entered for that mouse for the rest of the experiment. Body weight was monitored daily for Th1 transfer passive EAE.

#### Isolation and analysis of cells from the CNS in EAE-induced mice

Mononuclear cells were isolated from spinal cords and brains, tissues were digested with collagenase and DNase I (Roche) for 30 min at 37°C, and cells were separated on a 40–80% Percoll gradient by centrifugation at 500g for 30 min. Cells at the 40–80% interface were collected. Cells were also isolated from draining lymph nodes by passing through nylon mesh, followed by lysis of blood cells and washing with PBS. For intracellular cytokine staining, cells were stimulated with 80 nM PMA and 1 μM ionomycin in the presence of 3 μg/ml brefeldin A for 5 h and stained for CD4, IFN-γ, IL-17A and GM-CSF.

#### Tmem11 knockdown in human peripheral blood mononuclear cell analysis

For knocking down of Tmem11, cells were infected with lentiviruses encoding Tmem11-targeting shRNAs on days 1 and 2 using the spin-infection method. 48 h after infection, cells were selected with 1 μg/ml of puromycin for 2 days and then expanded for further 3 days with fresh media. On day 7, cells were activated with 1 μg/ml of OKT antibody and 1 μg/ml of anti-CD28 antibody for 5 h, intracellularly stained with anti-IFN-γ Ab-PE. For flow cytometry, the following human specific antibodies were used: anti-IFN-γ Ab-PE (45.B3, eBioscience).

#### RNA-seq analysis

For RNA-seq experiments naïve T cells from control and *Tmem11*^−/−^ mice were purified and stimulated on plate-coated anti-CD3 and anti-CD28 antibodies with 10 μg/ml anti-IL-4 antibody (Peprotech) and 2 ng/ml IL-12 for Th1 differentiation for 48 h. After 48 h, cells were taken off the plate and cultured in medium containing 10 U/ml recombinant human IL-2 (NCI Preclinical repository, for Th1 cells) for two days. At day 4 post isolation, ~3 million control and *Tmem11*^−/−^ Th1 cells were stimulated with anti-CD3 and anti-CD28 antibodies for 5 h and harvested in TriZol reagent (Invitrogen) for RNA preparation. RNA was isolated using RNA purification kit (ThermoFisher Scientific), quantified and used for purification of mRNA, cDNA synthesis and library preparation using TruSeq RNA Sample preparation kit (Illumina) following manufacturer’s instructions. The library of four samples was multiplexed and sequenced using Illumina Hiseq 2000 platform. After demultiplexing, each sample got ~30 million 50 bp single-end reads. Tophat was used to align reads to mouse genome GRCm38/mm10 with Ensembl 74 annotation with a cut-off of two maximum mismatches. Only uniquely mapped reads were used for downstream analysis. Htseq-count from HTSeq (version 0.5.3p9, http://www-huber.embl.de/users/anders/HTSeq/doc/overview.html) was used to quantify the number of reads aligned per gene with option intersection-nonempty and Ensembl 74 gene sets. Differential expression of genes was performed with DESeq v1.14. Genes with 0 reads across all samples were removed. Replicates were grouped and comparison was done with default DESeq parameters. Genes with adjusted p value ≤ 0.05 were considered differentially expressed. Normalized Count Values of candidates presented in Figure S2A were derived from independent RNA seq and DESeq2 analysis of WT naïve T cells cultured for 4 days under Th1- and pathogenic Th17-polarizing conditions and restimulated with anti-CD3 and anti-CD28 antibodies for 5 h.

#### Chromatin immunoprecipitation (ChIP)

For ChIP experiments, naïve T cells from control and *Tmem11*^−/−^ mice were purified and stimulated on plate-coated anti-CD3 and anti-CD28 antibodies with 10 μg/ml anti-IL-4 antibody (Peprotech) and 2 ng/ml IL-12 for Th1 differentiation for 48 h. After 48 h, cells were taken off the plate and cultured in medium containing 10 U/ml recombinant human IL-2 (NCI Preclinical repository, for Th1 cells) for two days. At day 4 post isolation, ~4 million control and *Tmem11*^−/−^ Th1 cells were stimulated with anti-CD3 and anti-CD28 antibodies for 5 h. ChIP assay was performed using the SimpleChIP Enzymatic Chromatin IP Kit (Cell Signaling Technology, Cat. #91820), according to the manufacturer’s instructions. Briefly, Th1 cells were cross-linked, lysed, and digested to generate DNA fragments. A total of 10 μg of digested chromatin was incubated overnight with 0.4 μg of H3K9Ac antibody or control IgG. Precipitated DNA was quantitated by real-time quantitative PCR analysis.

### QUANTIFICATION AND STATISTICAL ANALYSIS

#### Statistical analysis

Statistical analysis was carried out using two-tailed/unpaired Student’s t-test, one-way/two-way ANOVA, and Mann-Whitney *U* test as indicated. Differences were considered significant when *P* values were < 0.05. Differential gene expression was determined using the DESeq2 package (Bioconductor). DESeq2 determines p-values via the Wald test and are adjusted multiple testing using the Benjamini and Hochberg method. Genes with adjusted *P* value ≤ 0.05 were considered differentially expressed (DEGs).

## Supplementary Material

1

## Figures and Tables

**KEY RESOURCES TABLE:** 

REAGENT or RESOURCE	SOURCE	IDENTIFIER

Antibodies		

TMEM11	Proteintech	Cat# 16564-1-AP
MIC60	Proteintech	Cat# 10179-1-AP
MIC10	Bioss antibodies	Cat# BS-15029R
OXPHOS	Abcam	Cat# MS604-300
OPA1	BD biosciences	Cat# 612606
FLAG tag	Millipore Sigma	Cat# F3040
β-actin	Santa Cruz Biotechnology	Cat# sc-47778
anti-CD3 antibody	Bio X Cell	Cat# BE0001
anti-CD28 antibody	Bio X Cell	Cat# BE0015
anti-mIFN-γ antibody	Bio X Cell	Cat# BE0055
anti-mIL-4 antibody	Bio X Cell	Cat# BE0045
anti-hIFN-γ Ab-PE (45.B3)	ThermoFisher Scientific	Cat# 50-111-86
anti-IL-2 Ab-PE (MQ1-17H12)	ThermoFisher Scientific	Cat# 12-7029-81
anti-TNF Ab-APC (MAb11)	ThermoFisher Scientific	Cat# 17-7349-41
anti-T-bet Ab-APC (4B10)	ThermoFisher Scientific	Cat# 2124193
anti-IL-4 Ab-APC (11B11)	ThermoFisher Scientific	Cat# 17-7041-82
anti-IL-17A Ab-APC (eBio17B7)	ThermoFisher Scientific	Cat# 17-7177-81
anti-IFN-γ Ab-PE (XMG1.2)	ThermoFisher Scientific	Cat# 12-7311-82
anti-GM-CSF Ab-FITC (MP1-22E9)	ThermoFisher Scientific	Cat# 11-7331-82
anti-Foxp3 Ab-APC (FJK-16s)	ThermoFisher Scientific	Cat# 17-5773-82
anti-RORγT Ab-PE (AFKJS-9)	ThermoFisher Scientific	Cat# 12-6988-82
anti-CD4 Ab-PerCP (RM4-5)	ThermoFisher Scientific	Cat# 46-0042-82
anti-CD8b Ab-PE (H35-17.2)	ThermoFisher Scientific	Cat# 12-0083-82
anti-CD44 Ab-PerCP-Cy5.5 (IM7)	ThermoFisher Scientific	Cat# 45-0441-80
anti-CD62L Ab-APC (MEL-14)	ThermoFisher Scientific	Cat# 17-0621-81
anti-CD25 Ab-APC (PC61.5)	ThermoFisher Scientific	Cat# 17-0251-81
anti-CD69 Ab-PerCP-Cy5.5 (H1.2F3)	ThermoFisher Scientific	Cat# 45-0691-80
anti-CCL3 Ab-eFluor660 (DNT3CC)	Invitrogen	Cat# 50-7532-82
anti-CCL5 Ab-APC (21445)	Invitrogen	Cat# MA5-23557
anti-mouse H3K4ac Ab-FITC (EPR16596)	Abcam	Cat# ab176799
anti-mouse H3K9ac Ab-FITC (C5B11)	Cell Signaling	Cat# 9649
anti-mouse histone H3 Ab-PE (D1H2)	Cell Signaling	Cat# 82241S
anti-mouse H3K27ac Ab- FITC (D5E4)	Cell Signaling	Cat# 15485S
anti-mouse H3K27me3 Ab (C36B11)	Cell Signaling	Cat# 9733
anti-mouse H3K4me3 Ab-PE (D1A9)	Cell Signaling	Cat# 55800S
OKT3	BD	Cat# 555330
anti-hCD28 antibody	Bio X Cell	Cat# BE0248
Goat anti-hamster antibody	MP Biomedicals	Cat# 0855397
anti-Acetyl-Histone H3 (Lys9) antibody (C5B11)	Cell Signaling	Cat# 9649
Anti-Glucose Transporter GLUT1 antibody	Abcam	Cat# ab115730

Bacterial and virus strains		

*E. coli* DH5α	Thermo Fisher Scientific	Cat# 18265017

Chemicals, peptides, and recombinant proteins		

Fura 2-AM	Thermo Fisher Scientific	Cat# F1221
Brefeldin A	Thermo Fisher Scientific	Cat# 00-4506-51
Thapsigargin	EMD Millipore	Cat# 80055-474
Phorbol 12-myristate 13-acetate (PMA)	EMD Millipore	Cat# 5.00582.0001
lonomycin	EMD Millipore	Cat# 407951
Polybrene	Millipore Sigma	Cat# TR-1003
Puromycin	Invivogen	Cat# ant-pr-1
Blasticidin A	Invivogen	Cat# ant-bl-05
Poly-D-Lysine	Thermo Fisher Scientific	Cat# A003E
L-glutamine	Gibco	Cat# 25-005-CI
Sodium Pyruvate	Fisher Scientific Company	Cat# MT25000CI
oligomycin	Abcam	Cat# ab141829
protonophore carbonyl cyanide-4-(trifluoromethoxy)-phenylhydrazone	Cayman Chemical	Cat# 370-86-5
rotenone	AdipoGen	Cat# AG-CN2-0516
antimycin A	Sigma	Cat# A8674
2-NBDG	Invitrogen	Cat# N13195
BODIPY 493/503	Invitrogen	Cat# D3922
MitoSOX Red	Invitrogen	Cat# M36008
TTFA	Sigma	Cat# T27006
TOFA	Sigma	Cat# T27006
Tetramethylrhodamin-Ethylester	Thermo Fisher Scientific	Cat# T669
MitoTracker^™^ Green FM	Invitrogen	Cat# M7514
DNase I	Worthington Biochem	Cat# LS002007
IL-12	Peprotech	Cat# 210-12
IL-4	Peprotech	Cat# 214-14
IL-2	Peprotech	Cat# 200-02
IL-6	Peprotech	Cat# 200-06
TGF-β	R&D Systems	Cat# 7666-MB-005/CF
mIL-23	R&D Systems	Cat# 1887-ML-010/CF
mIL-1β	R&D Systems	Cat# 401-ML-010/CF
Fixable Viability Dye eFluor^™^ 780	eBioscience	Cat# 65-0865-14
TRIzol	Thermo Fisher Scientific	Cat# 15596026
protease inhibitor cocktail	Roche	Cat# 04693132001
MOG_35–55_ peptide	Genscript	Cat# sc1848
Complete Freund’s Adjuvant	BD	Cat# 90003-746
Mycobacterium tuberculosis H37Ra	BD	Cat# 90002-208
pertussis toxin	List Biological Laboratories	Cat# 102946-454
Percoll	Sigma	Cat# P1644

Critical commercial assays		

MagniSort human naïve CD4^+^ T cell enrichment kit	Thermo Fisher Scientific	Cat# 8804-6814-74
MagniSort mousenaïve CD4^+^ T cell enrichment kit	Thermo Fisher Scientific	Cat# 8804-6824-74
CellTrance Violet Cell Proliferation kit	Thermo Fisher Scientific	Cat# C34557
FOXP3/Transcription Factor staining Buffer set	Thermo Fisher Scientific	Cat# 2229155
Direct-zol RNA isolation kit	Zymo Research	Cat# R2062
Perfecta SYBR SuperMix-IQ	Quantabio	Cat# 101414-144
qScript^™^ cDNA SuperMix	Quantabio	Cat# 101414-108
acetyl-coenzyme A assay kit	Sigma	Cat# MAK039
citrate assay kit	Sigma	Cat# MAK057
GSH/GSSG-Glo^™^ Assay kit	Promega	Cat# V6611
ATPlite 1step Luminescence Assay kit	PerkinElmer	Cat# 6016736
SimpleChIP Enzymatic Chromatin IP Kit	Cell Signaling Technology	Cat# 91820
TruSeq RNA Sample preparation kit	Illumina	N/A

Experimental models: Cell lines		

HEK293T	ATCC	Cat# CRL-3216

Recombinant DNA		

pMD2.G	Addgene	Addgene plasmid # 12259
psPAX2	Addgene	Addgene plasmid # 12260
MO70-C-FLAG-hTmem11	This paper	Details in Table S1
hTmem11 sh#1	Sigma	Cat# TRCN0000273652
hTmem11 sh#2	Sigma	Cat# TRCN0000008147

Software and algorithms		

Slidebook software	Intelligent Imaging Innovations, Inc.	N/A
OriginPro	Originlab	N/A
Image J	NIH	N/A
Zeiss LSM 880 confocal microscope	Zeiss	N/A
Zen Black software	Zeiss	N/A
Aivia v.10 software	Leica Microsystems	N/A
JEOL 100CX transmission electron microscope	Advanced Microscopy Techniques Corporation	N/A
Illumina Hiseq 2000 platform	Illumina	N/A
DESeq2 v1.14	Bioconductor	N/A

Deposited data		

RNA-seq data (public)	Gene Expression Omnibus	GSE283950

Other		

iCycler IQ5 system	Bio-Rad	N/A
BD Fortessa flow cytometer	BD Biosciences	N/A
Aurora Flow Cytometer	Cytek	N/A
AMT digital camera	Advanced Microscopy Techniques Corporation	N/A
LAS-3000 LCD camera	FujiFilm	N/A
Q Exactive mass spectrometer	ThermoFisher Scientific	N/A
Seahorse XFe96 Bioanalyzer	Agilent	N/A

## Highlights

- A mitochondrial inner membrane protein, TMEM11, regulates Th1 responses
- *Tmem11*^−/−^ Th1 cells show altered mitochondrial cristae morphology and high mtROS
- Increased mtROS impaired histone acetylation and enhanced fatty acid synthesis
- Cristae integrity can influence cellular metabolism and chromatin modification
