# Supplementary material for: Mitochondrial reactive oxygen species regulate acetyl-CoA flux between cytokine production and fatty acid synthesis in effector T cells
Source: Cell Rep. Author manuscript; Available in PMC 2025 Apr 18. (PMC12007815; doi:10.1016/j.celrep.2025.115430)
Supplement: 1 [file NIHMS2069125-supplement-1.pdf]

Cell Reports, Volume *44*

Supplemental information

Mitochondrial reactive oxygen species regulate acetyl-CoA flux between cytokine production and fatty acid synthesis in effector T cells

Beibei Wu, Jin Seok Woo, Spyridon Hasiakos, Calvin Pan, Shawn Cokus, Cristiane Benincá, Linsey Stiles, Zuoming Sun, Matteo Pellegrini, Orian S. Shirihai, Aldon J. Lusis, Sonal Srikanth, and Yousang Gwack

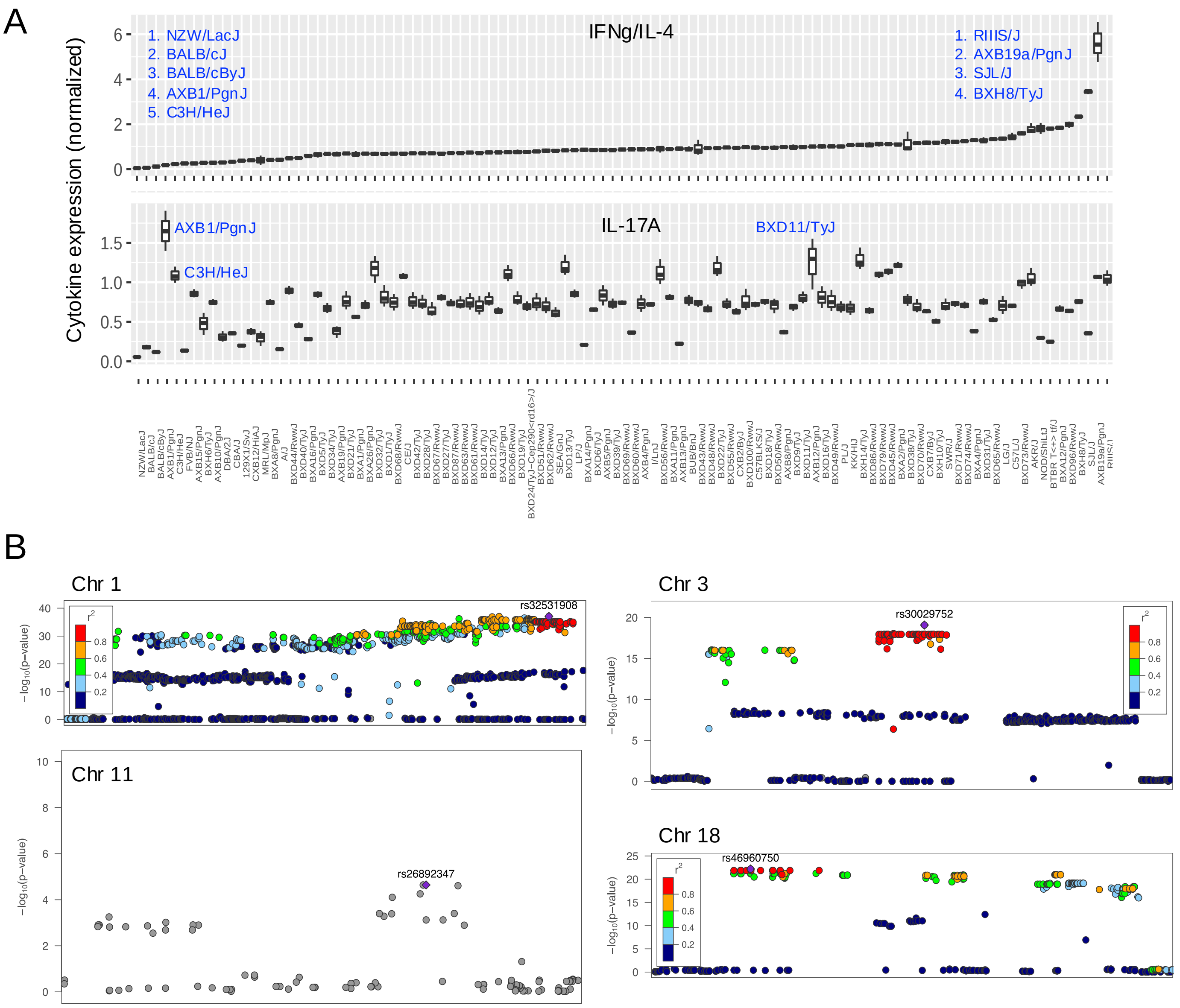

Supplementary Figure 1

Supplementary Figure 1. Mouse panel screening.

A, Distribution of 120 inbred mouse strains of the HMDP for expression of indicated cytokines from splenocytes. Splenocytes were isolated from 3-4 mice of each strain and cultured under non-polarizing (only anti-CD3 antibodies) or Th17-polarizing conditions (anti-CD3 antibodies together with IL-6 [30 ng/ml] and IL-23 [10 ng/ml]) to check cytokine levels (the ratio of IFNγ to IL-4 and IL-17A) in $CD4^+$ cells by intracellular staining. Splenocytes were cultured for 3 days and re-stimulated with PMA (80 nM) plus ionomycin (1 μM) prior to staining. Y-axis indicates fold change in expression of indicated cytokines normalized to those in cells isolated from C57BL/6J mice. Individual mouse strains (x-axis) are arranged based on increasing ratio of IFNγ/IL-4 expression (top). Bottom panel shows IL-17A expression in the same strains.

B, Locus plot for genome-wide significant association at chromosomes 1, 3, 11 and 18. Representation of the association strength (-log10 p value, y axis) versus the genomic location (x axis) around the most significant SNPs, indicated with a red circle. Other SNPs in the region are color coded to reflect their linkage disequilibrium (LD) scores with the top SNP, as in the left inset (taken from pairwise r2 values calculated on Sardinian haplotypes).

Related to **Figure 1**.

A

gene_symbol	gene_description	chr	Th1 (counts)	Th17 (counts)
Fbxo28	F-box protein 28 [Source:MGI Symbol;Acc:MGI:1261890]	1	1299.52	1419.26
Trp53bp2	transformation related protein 53 binding protein 2 [Source:MGI Symbol;Acc:MGI:2138319]	1	749.82	636.74
Capn2	calpain 2 [Source:MGI Symbol;Acc:MGI:88264]	1	4604.17	3456.34
Ccny	cyclin Y [Source:MGI Symbol;Acc:MGI:1915224]	18	1986.86	2124.18
Thoc1	THO complex 1 [Source:MGI Symbol;Acc:MGI:1919668]	18	2194.7	1987.73
Usp14	ubiquitin specific peptidase 14 [Source:MGI Symbol;Acc:MGI:1928898]	18	2132.52	1486.67
Flcn	folliculin [Source:MGI Symbol;Acc:MGI:2442184]	11	1011.67	730.96
Tmem11	transmembrane protein 11 [Source:MGI Symbol;Acc:MGI:2144726]	11	1011.33	1447.51
Guk1	guanylate kinase 1 [Source:MGI Symbol;Acc:MGI:95871]	11	491.91	468.86
Zkscan17	zinc finger with KRAB and SCAN domains 17 [Source:MGI Symbol;Acc:MGI:2679270]	11	1210.03	1809.88
Llgl1	lethal giant larvae homolog 1 (Drosophila) [Source:MGI Symbol;Acc:MGI:102682]	11	1074.15	1001.55
Jmjd4	jumonji domain containing 4 [Source:MGI Symbol;Acc:MGI:2144404]	11	666.4	434.69
Dhrs7b	dehydrogenase/reductase (SDR family) member 7B [Source:MGI Symbol;Acc:MGI:2384931]	11	462.22	452.7

B

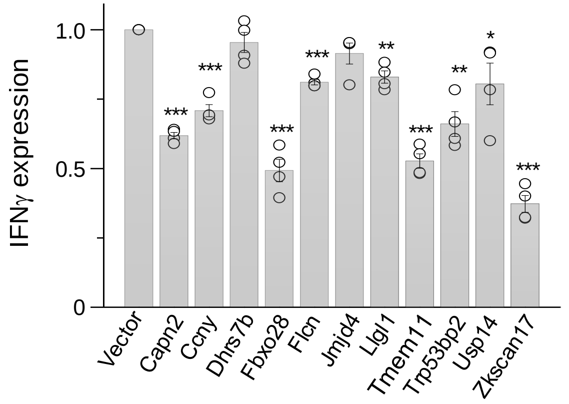

C

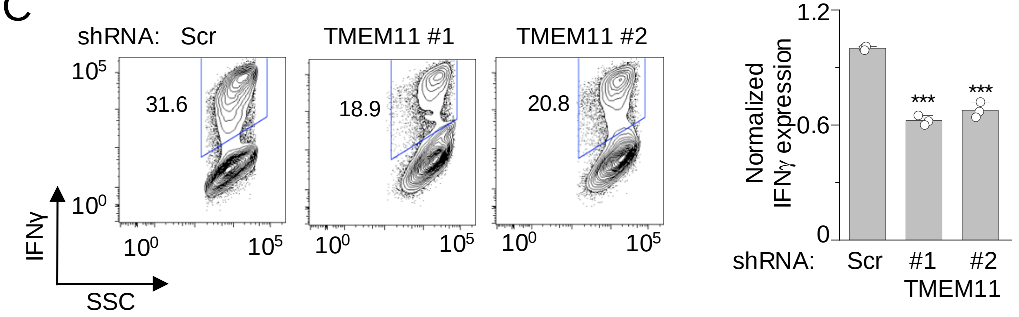

Supplementary Figure 2

Supplementary Figure 2. Screening of candidates from mouse panel screen for their potential role in effector T cell functions.
A, Expression of candidate genes expressed as normalized counts (DESeq2) derived from RNA-seq of wild-type Th1 and Th17 cells. Gene list was curated from those located within the chromosomal loci uncovered from HMDP analysis and showing significant expression in T cells (normalized counts > 100).
B, IFNγ expression by WT $CD4^+$ T cells cultured under Th1-polarizing conditions for 4 days and transduced with retroviruses to overexpress indicated genes. The cells were re-stimulated with anti-CD3 and anti-CD28 antibodies and processed for cytokine staining. Data are normalized to those obtained with cells transduced with empty vector.
C, IFNγ expression in human $CD4^+$ T cells cultured under Th1-polarizing conditions for 6 days after transduction with lentiviruses encoding shRNAs targeting TMEM11 transcripts. The cells were re-stimulated with anti-CD3 and anti-CD28 antibodies and processed for cytokine staining. Data are normalized to those obtained with cells transduced with lentiviruses encoding scrambled shRNA (Src).
Individual points in bar graphs in panels b and c show data from independent replicates. Data represent means ± s.e.m., significance was determined by unpaired two-tailed *t*-test. *$P < 0.05$, ** $P < 0.005$, and *** $P < 0.0005$.
Related to **Figure 1**.

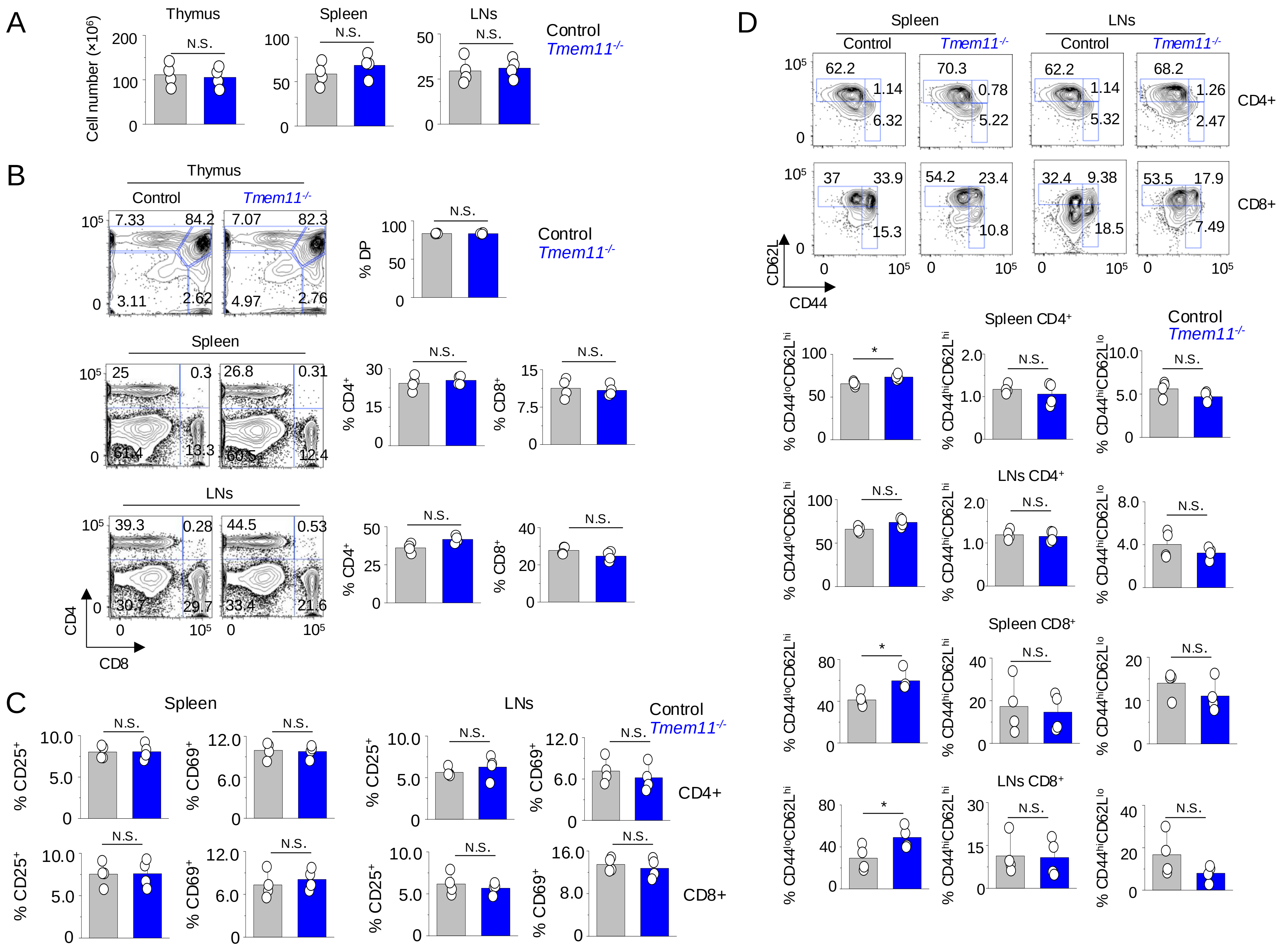

Supplementary Figure 3

Supplementary Figure 3. Deletion of *Tmem11* does not affect T cell development.
A, Total cell numbers from the thymi, spleen, and lymph nodes of control and *Tmem11*$^{-/-}$ mice.
B, Representative flow plots (left) showing frequency of $CD4^+$ and $CD8^+$ populations and bar graphs (right) from lymphoid organs of control and *Tmem11*$^{-/-}$ mice.
C, Bar graphs showing frequencies of $CD25^+$ and $CD69^+$ cells among $CD4^+$ and $CD8^+$ T cell populations in the spleen and lymph nodes (LN) of control and *Tmem11*$^{-/-}$ mice.
D, Representative flow plots showing frequencies of naïve, central-, and effector memory $CD4^+$ and $CD8^+$ T cell populations in the spleen and lymph nodes of control and *Tmem11*$^{-/-}$ mice as judged by surface staining for CD62L and CD44.
In the bar graphs in all the panels, each symbol represents data obtained from an independent animal. $*P < 0.05$, N.S., not significant.
Related to **Figure 1**.

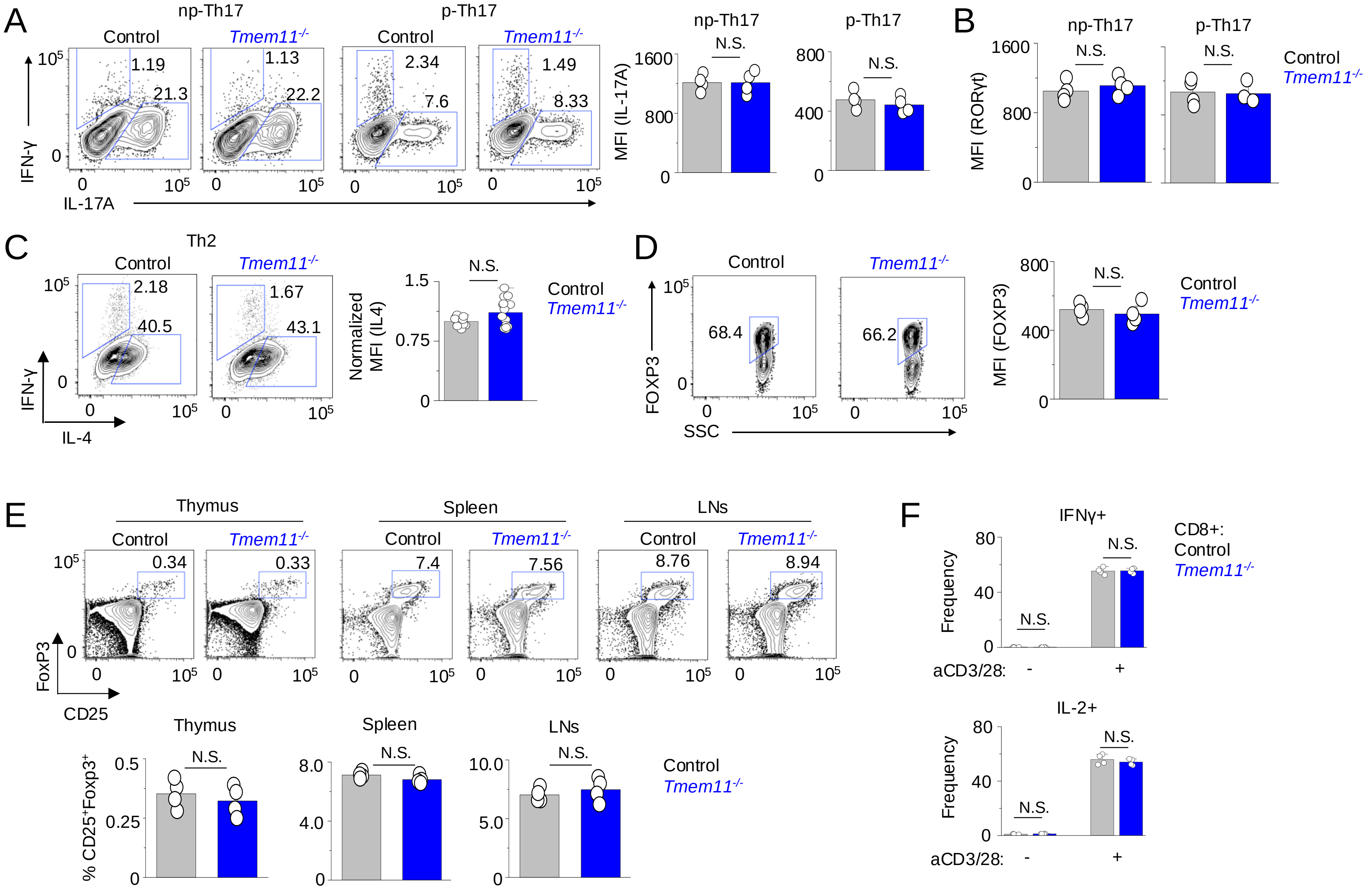

Supplementary Figure 4

Supplementary Figure 4. Deletion of *Tmem11* does not affect Th17, Th2, Treg, and CD8$^+$ T cell functions.
A, Representative flow plots (left) showing frequencies and bar graph (right) showing mean fluorescence intensities of IL-17A expression in CD4$^+$ T cells from control and *Tmem11*$^{-/-}$ mice cultured under non-pathogenic - (np-Th17) and pathogenic - Th17-polarizing (p-Th17) conditions for 4 days and re-stimulated with anti-CD3 and anti-CD28 antibodies.
B, MFI of RORγt in non-pathogenic or pathogenic Th17 cells from control and *Tmem11*$^{-/-}$ mice, cultured as described above (without re-stimulation).
C, Representative flow plots (left) showing frequencies and bar graph (right) showing mean fluorescence intensities of IL-4 expression in CD4$^+$ T cells from control and *Tmem11*$^{-/-}$ mice cultured under Th2-polarizing conditions for 4 days and re-stimulated with anti-CD3 and anti-CD28 antibodies.
D, Representative flow plots (left) showing frequencies and bar graph (right) showing mean fluorescence intensity of FOXP3$^+$ cells, among iTregs differentiated from naïve CD4$^+$ T cells for 4 days from control and *Tmem11*$^{-/-}$ mice.
E, Representative flow plots (top) and bar graphs (below) showing frequencies of natural regulatory T cells (CD4$^+$FoxP3$^+$) from the thymi, spleen and lymph nodes of control and *Tmem11*$^{-/-}$ mice.
F, Bar graphs showing frequencies of IFNγ or IL-2-expressing CD8$^+$ T cells from control and *Tmem11*$^{-/-}$ mice cultured for 4 days and re-stimulated with anti-CD3 and anti-CD28 antibodies before intracellular staining.
For all the panels, each symbol represents data obtained from an independent animal. N.S., not significant.
Related to **Figure 1**.

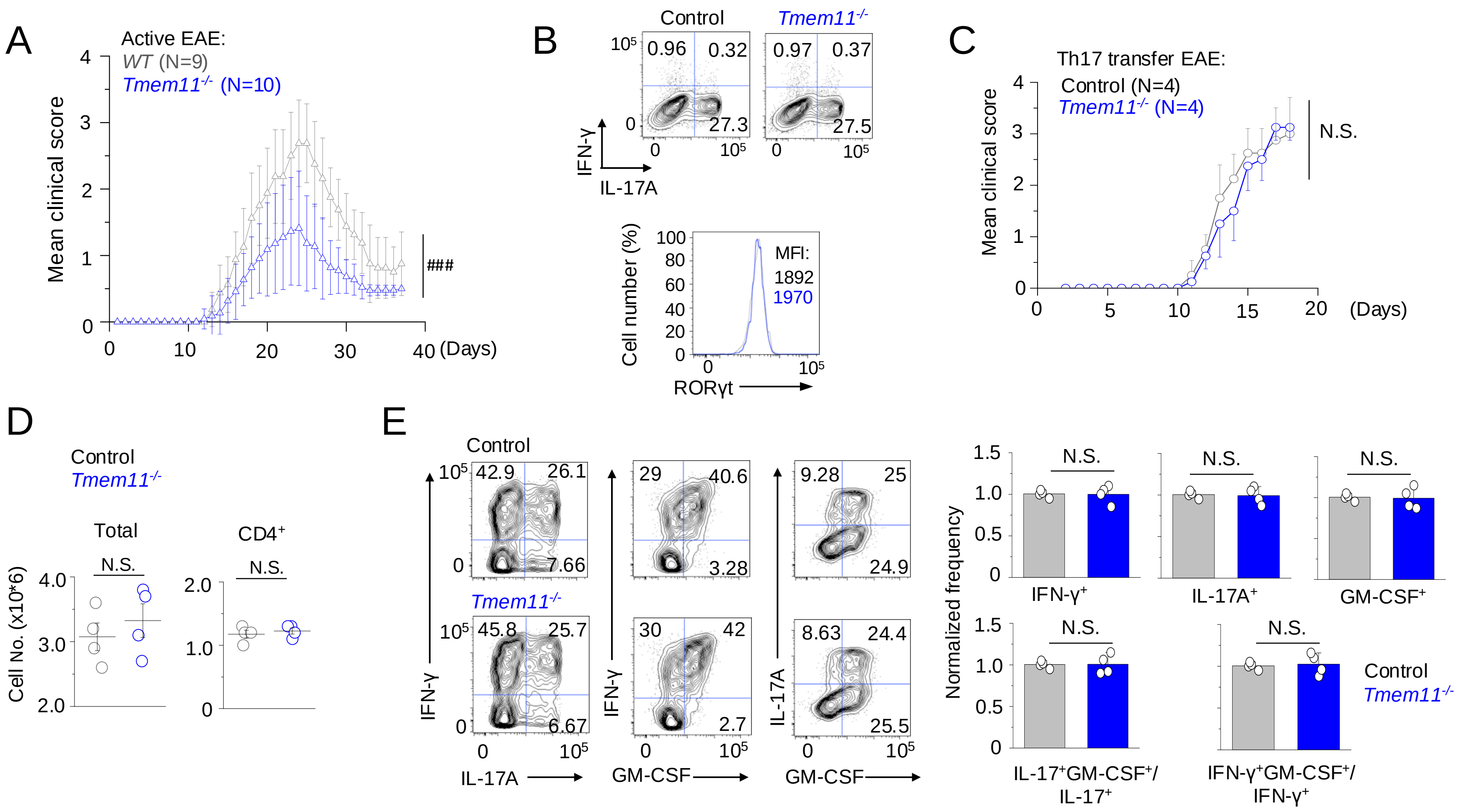

Supplementary Figure 5

Supplementary Figure 5. Loss of *Tmem11* does not influence Th17 cell function *in vivo*.
A, Time course of the mean clinical score of EAE in control and *Tmem11*$^{-/-}$ mice. The line graph shows mean ± s.e.m. from the indicated number of animals pooled from 3 independent experiments.
B, Representative flow plots showing IFNγ , IL-17A and RORγt expression in CD4^{+} cells from the draining lymph nodes of control or *Tmem11*$^{-/-}$ mice injected with MOG peptide for EAE induction and cultured under Th17 expansion conditions (30 ng/ml IL-6 and 10 ng/ml IL-23) for 72 h.
C, Time course of the mean clinical score of EAE in *Rag2*$^{-/-}$ recipients of control or *Tmem11*$^{-/-}$ draining lymph node cells cultured under Th17 expansion conditions. The line graph shows mean ± s.e.m. from the indicated number of animals from one experiment.
D, Scatter plots showing the numbers of total mononuclear (left) or CD4^{+} T cells (right) isolated from the CNS of recipients of control or *Tmem11*$^{-/-}$ cells at the peak of EAE.
E, Representative flow plots showing the cytokine expression profile of CD4^{+} T cells from the CNS of recipients of control or *Tmem11*$^{-/-}$ cells at the peak of the disease (left). Bar graphs show average (± s.e.m.) of normalized frequency of IFNγ^{+}, IL-17A^{+}, and GM-CSF^{+} cells (right).
Individual points in bar graphs in panels d and e show data from independent animals. Data represent means ± s.e.m., significance was determined by unpaired two-tailed *t*-test (d and e) and Mann-Whitney *U* test (a and c). $^{###}p<0.0001$, N.S. not significant.
Related to **Figure 1**.

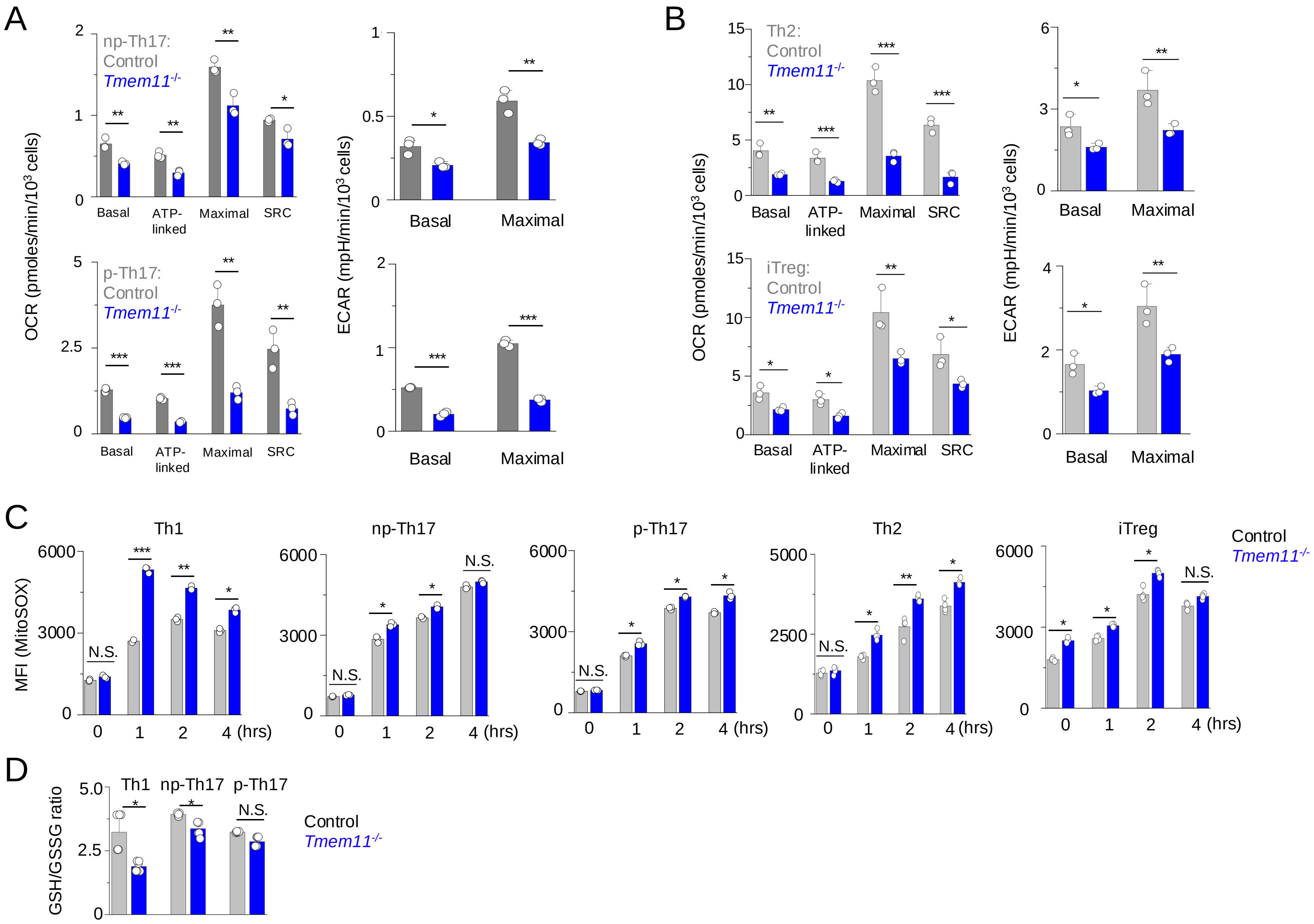

Supplementary Figure 6

Supplementary Figure 6. Measurement of reactive oxygen species and oxidative stress levels in *Tmem11*$^{-/-}$ effector T cells.
A, Oxygen consumption rate (OCR - left) and extracellular acidification rate (ECAR - right) measurements in control and *Tmem11*$^{-/-}$ T cells cultured under non-pathogenic and pathogenic Th17-polarizing conditions. Th17 cells were stimulated with anti-CD3 and anti-CD28 antibodies for 2 h before measurements. Data are average ± s.e.m from cells cultured from three independent animals.
B, OCR and ECAR measurements in control and *Tmem11*$^{-/-}$ T cells cultured under Th2 and iTreg-polarizing conditions.
C, Measurement of reactive oxygen species (ROS) levels in WT and *Tmem11*$^{-/-}$ effector T cells after re-stimulation with anti-CD3 and anti-CD28 antibodies for indicated times.
D, Cellular oxidative stress levels in WT and *Tmem11*$^{-/-}$ cells cultured under Th1- and Th17-polarizing conditions as determined by measuring the GSH/GSSG ratio after 2 h of re-stimulation with anti-CD3 and anti-CD28 antibodies.
Individual points in bar graphs in c and d show data from independent technical replicates from two biological samples. Datarepresent means ± s.e.m., significance was determined by unpaired two-tailed *t*-test. *$P < 0.05$, ** $P < 0.005$, and *** $P < 0.0005$, N.S. not significant.
Related to **Figures 3 and 4**.

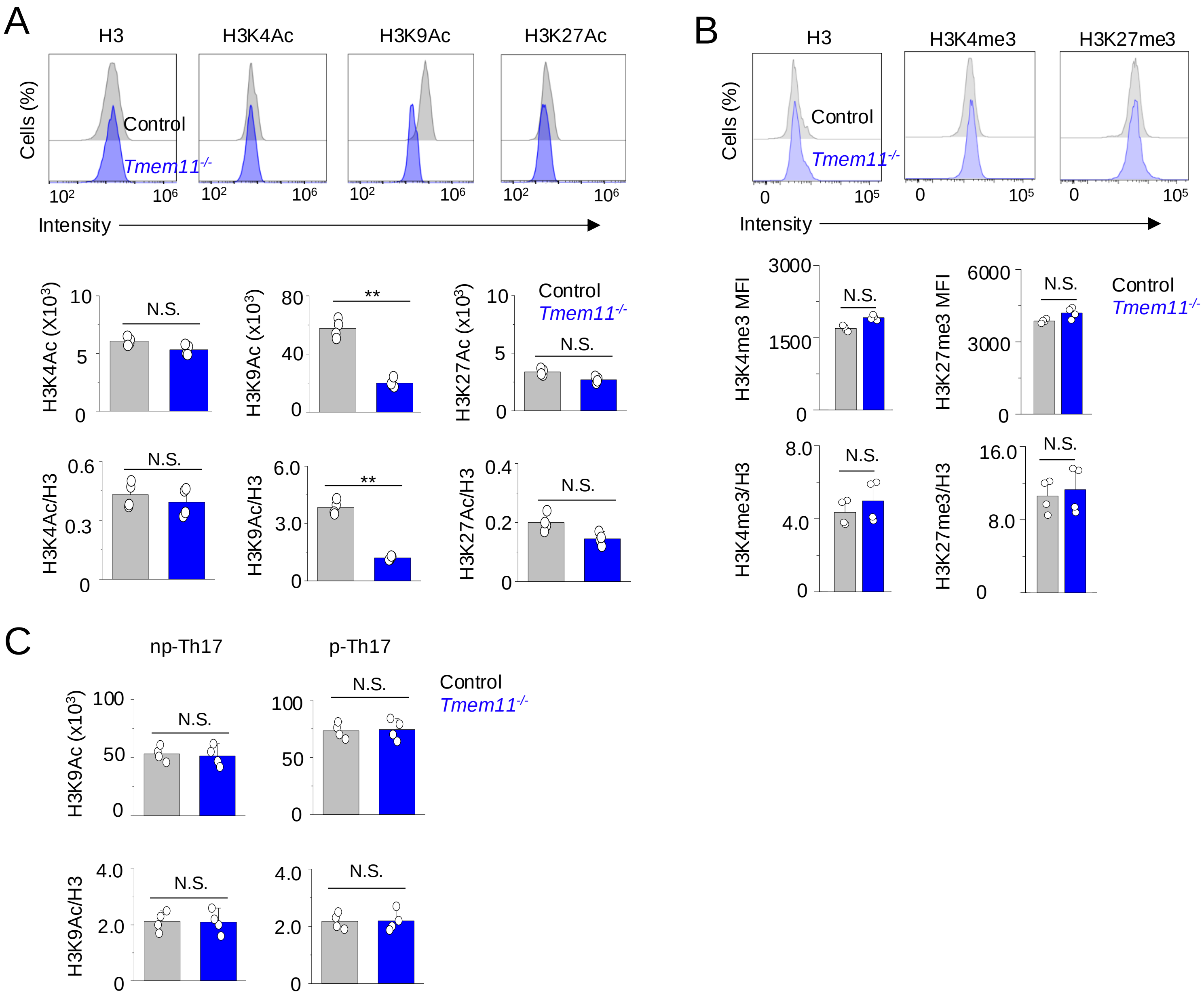

Supplementary Figure 7

Supplementary Figure 7. Histone acetylation and methylation levels in *Tmem11*$^{-/-}$ cells.
A, Measurement of cellular H3K4Ac, H3K9Ac and H3K27Ac levels in WT and *Tmem11*$^{-/-}$ cells cultured under Th1 cell-polarizing conditions after re-stimulation with anti-CD3 and anti-CD28 antibodies for 5 h.
B, Measurement of cellular H3K4m3 and H3K27m3 levels in WT and *Tmem11*$^{-/-}$ cells cultured under Th1 cell-polarizing conditions after re-stimulation with anti-CD3 and anti-CD28 antibodies for 5 h.
C, Measurement of cellular H3K9Ac levels in WT and *Tmem11*$^{-/-}$ naïve $CD4^+$ T cells cultured under non-pathogenic or pathogenic Th17 cell-polarizing conditions after re-stimulation with anti-CD3 and anti-CD28 antibodies for 5 h.
Bar graphs in all the panels show means ± s.e.m from 2 biological samples with multiple technical replicates; significance was determined by unpaired two-tailed t-test. **P < 0.005, N.S. not significant.
Related to **Figure 5**.

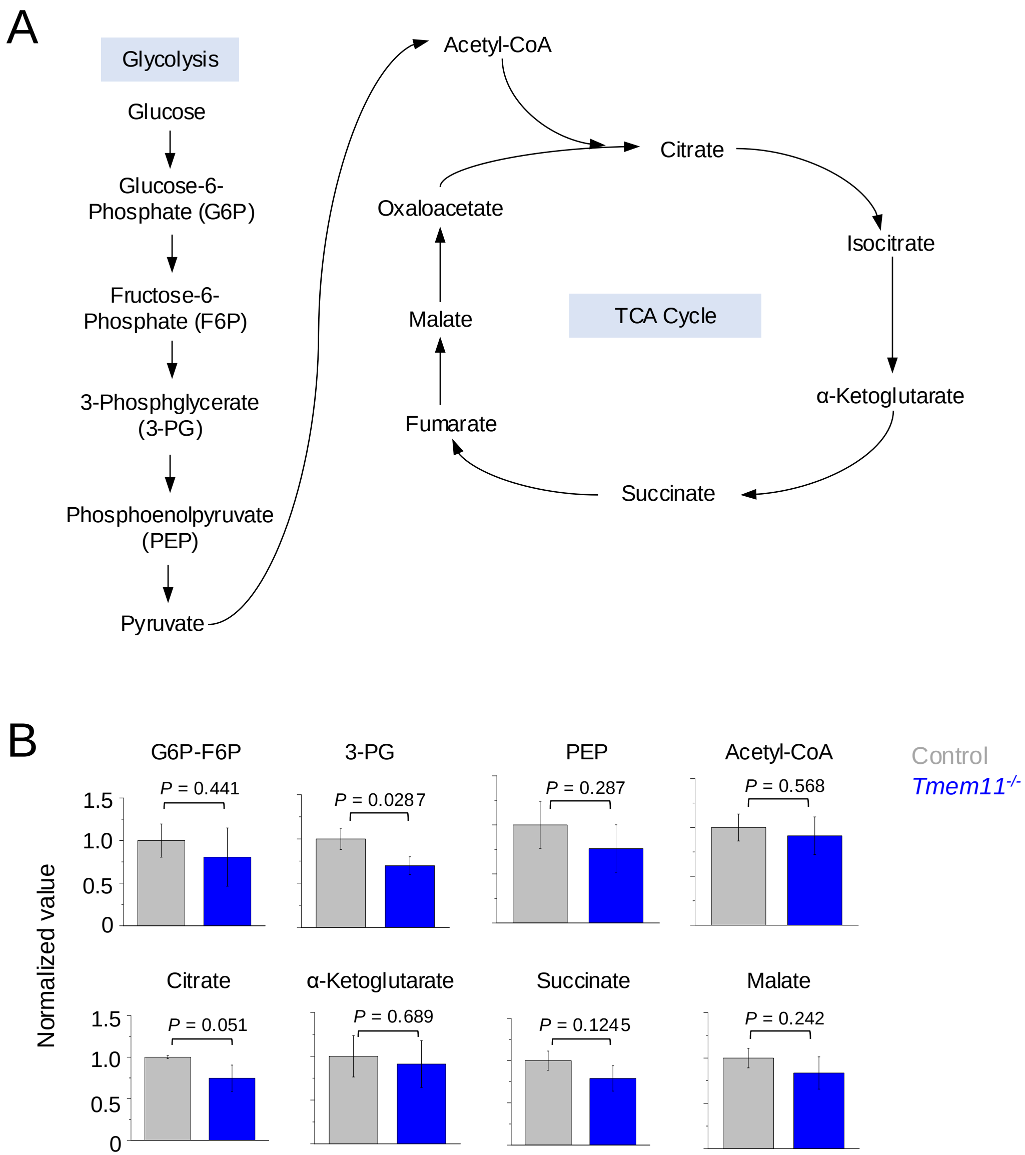

Supplementary Figure 8

Supplementary Figure 8. TCA metabolites analysis between WT and *Tmem11*$^{-/-}$ Th1 cells.
A, Schematic showing steps in glycolysis and TCA cycle.
B, Metabolomic analysis of control and *Tmem11*$^{-/-}$ Th1 cells after re-stimulation with anti-CD3 and anti-CD28 antibodies for 5 h showing levels of G6P-F6P, 3-phosphoglycerate (3-PG), phosphoenolpyruvate (PEP), Acetyl-CoA, citrate, α-ketoglutarate, succinate, and malate. Values were normalized to those of control cells.
Bar graphs show data from technical replicates from three biological samples. Data represent means ± s.e.m., significance was determined by unpaired two-tailed *t*-test.
Related to **Figure 5.**

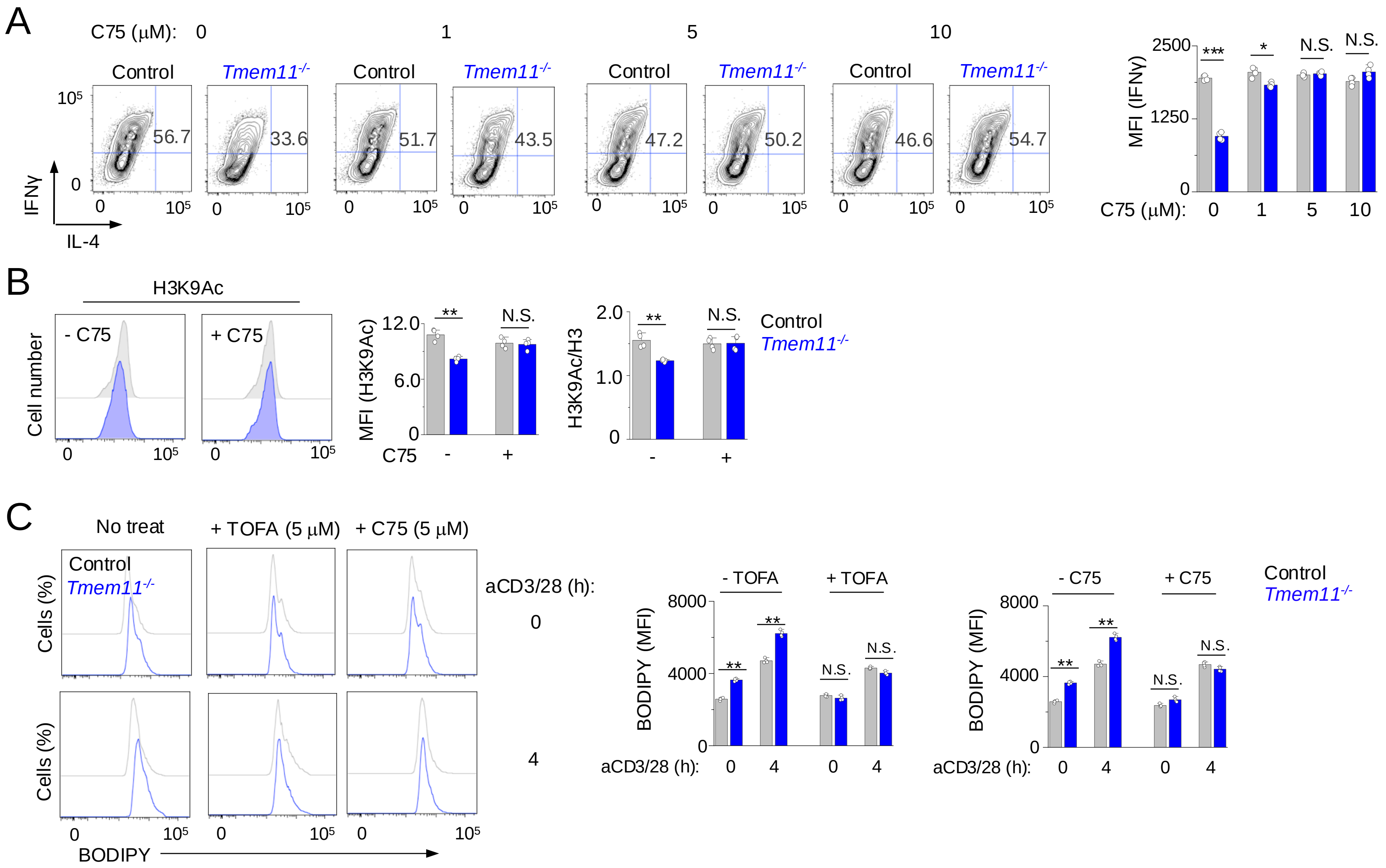

Supplementary Figure 9

Supplementary Figure 9. TMEM11 deficiency decreases H3K9Ac levels and increases fatty acid synthesis.
A and B, Measurement of intracellular cytokine expression (**A**) and H3K9Ac levels (**B**) in WT and *Tmem11*$^{-/-}$ cells cultured under Th1-polarizing conditions and treated with 5 μM C75 overnight and during re-stimulation with anti-CD3 and anti-CD28 antibodies for 5 h.
C, Representative flow plots (left) and bar graph (right) showing measurement of neutral lipid levels in WT and *Tmem11*$^{-/-}$ cells cultured under Th1-polarizing conditions and re-stimulated for the indicated times. Cells were treated with 5 μM TOFA or C75 overnight and during re-stimulation.
Individual points in bar graphs show data from independent technical replicates from at least two biological samples. Data represent means ± s.e.m., significance was determined by unpaired two-tailed *t*-test. *$P < 0.05$, ** $P < 0.005$, and *** $P < 0.0005$, N.S. not significant.
Related to **Figure 5**.

Supplementary Table

Table S1. List of primers used in this study.

Gene name	Forward Primer	Reverse Primer	Comments
Ccl3	CTGCCTGCTGCTTCTCCTAC	GTTCCTCGCTGCCTCCAA	qPCR primers
Ccl4	GCTTCCTCGCAACTTTGTGG	TCACTGGGATCAGCACAGAC	qPCR primers
Ccl5	TCATTGCTACTGCCCTCTGC	TACTCCTTGATGTGGGCACG	qPCR primers
Ccl9	CCCTCTCCTTCCTCATTCTTACA	AGTCTTGAAAGCCCATGTGAAA	qPCR primers
36b4	AGATTCGGGATATGCTGTTGGC	TCGGGTCCTAGACCAGTGTTC	qPCR primers
mtDNA Region 1	TGAACGGCTAAACGAGGGTC	AGCTCCATAGGGTCTTCTCGT	qPCR primers
mtDNA Region 2	CAGTCCCCTCCCTAGGACTT	ACCCTGGTCGGTTTGATGTT	qPCR primers
mtDNA Region 3	TAATCGCACATGGCCTCACA	GAAGTCCTCGGGCCATGATT	qPCR primers
Ifng	ACTGGCAAAAGGATGGTG	GTTGCTGATGGCCTGATT	qPCR primers
Tbx21	CAACAACCCCTTTGCCAAAG	TCCCCCAAGCAGTTGACAGT	qPCR primers
Cxcr3	TGCTAGATGCCTCGGACTTT	ATAAGACGGATGGCCTTGTG	qPCR primers
Ccr5	CGAAAACACATGGTCAAACG	TTCCTACTCCCAAGCTGCAT	qPCR primers
Gapdh	TGG AGA TTG TTG CCA TCA ACG ACC C	TAG ACT CCA CGA CAT ACT CAG CAC CG	qPCR primers

Stat4	AACCCTCCATCTGTCACTTT	CCATGATGTACCCATCAATC	qPCR primers
Eomes	TGTTTTCGTGGAAGTGGTTCTGGC	AGGTCTGAGTCTTGGAAGGTTCATTC	qPCR primers
Stat1	CGCGCATGCAAGTGGCATATAACT	AAGCTCGAACCACTGTGACATCCT	qPCR primers
Ifng	AGATGCTGACTCAAGACCCC	TTCATCCCTGCCTTTCCTGT	ChIP qPCR primers
Ccl3	GAGAGTTTCCGTTCCATGGC	ATGAGCAGGAGGAGGTAGGA	ChIP qPCR primers
Ccl4	GGCACTGCTCTGTCTTTTCC	CCCTAGACCTCACACTGCAA	ChIP qPCR primers
Ccl5	CAGGACAGACTTGGGACCAT	GTCCAGGTGAGAGTGCTGAT	ChIP qPCR primers
Ccl9	CTTCCCACTGCACACTGTTC	TGGCCCGGTCTTGCTATTTA	ChIP qPCR primers
h*TMEM11*	CCCTCGAGATGGCCGCTTGGGGAAGG	CGGAATTCTACGGCATAGAGTTCGTA	Overexpression
h*CAPN2*	CC CTC GAG ATG GCG GGC ATC GCG ATA	GC GTT AAC GAG TAC TGA AAA ACT CAG	Overexpression
h*CCNY*	CC CTC GAG ATG GGG AAC ACA ACT TCG	GC AGA TCT GGA GAT GAT GGC TGG AGA	Overexpression

h*DHRS7b*	CC CTC GAG ATG ATC TCT CCG TCC TTT	GC GAA TTC GGA GCT CTT GGA TTT CCG	Overexpression
h*FBXO28*	GC AGA TCT ATG GCG GCG GCG TCT GAG	GA GAA TTC CTT CCT ATT CCG AAG TCG	Overexpression
h*FLCN*	CC CTC GAG ATG AAC GCC ATA GTC GCC	GC GAA TTC GCT CCG TGA CTC TGT AGC	Overexpression
h*JMJD4*	GCGAATTCATGGATCGGGAGACACGC	GCCTCGAGCTAAAGGGCCTCTGCAGC	Overexpression
h*LLGL1*	GC GAA TTC ATG ATG AAG TTT CGG TTC	GC GTT AAC TTT AAT CAG AAG GGT GTA	Overexpression
h*TRP53BP2*	CC CTC GAG ATG CGG TTC GGG TCC AAA	GC GAA TTC GGC CAA GCT CCT TTG TCT	Overexpression
h*USP14*	CC CTC GAG ATG CCA CTC TAC TCT GTT	GC GAA TTC CTG TTC ACT TTC TTC TTC	Overexpression
h*ZKSCAN17*	CC CTC GAG ATG CCC ACA GCC CTG TGC	GC GTT AAC GTA TGA GTT GAG AGC CTG	Overexpression
